# Chalcomoracin is a potent anticancer agent acting through triggering Oxidative stress via a mitophagy- and paraptosis-dependent mechanism

**DOI:** 10.1038/s41598-018-27724-3

**Published:** 2018-06-22

**Authors:** Haote Han, Chih-Chien Chou, Ruyi Li, Jiangyun Liu, Lin Zhang, Wei Zhu, Jin Hu, Bingxian Yang, Jingkui Tian

**Affiliations:** 1 College of Biomedical Engineering & Instrument Science, Zhejiang University, Hangzhou, 310027 P.R. China; 2 Zhejiang-Malaysia Joint Research Center for Traditional Medicine, Zhejiang University, Hangzhou, 310027 P.R. China; 3 Department of Radiation Oncology, Stanford University School of Medicine, Stanford, CA United States of America; 4 A College of Pharmaceutical Sciences, Soochow University, Suzhou, 215123 P.R. China

## Abstract

Chalocomoracin (CMR), one of the major secondary metabolites found in fungus-infected mulberry leaves, is a potent anticancer agent. However, its anticancer mechanism remains elusive. Here, we demonstrated the potent anti-tumor activity and molecular mechanism of CMR both *in vitro* and *in vivo*. We showed for the first time that CMR treatment markedly promoted paraptosis along with extensive cytoplasmic vacuolation derived from the endoplasmic reticulum, rather than apoptosis, in PC-3 and MDA-MB-231cell lines. Additional studies revealed that ectopic expression of Myc-PINK1 (PTEN-induced kinase 1), a key regulator of mitophagy, rendered LNCap cells susceptible to CMR-induced paraptosis, suggesting that the mitophagy-dependent pathway plays a crucial role in inducing paraptosis by activating PINK1. CMR treatment directly upregulated *PINK1* and downregulated *Alix* genes in MDA-MB-231 and PC-3 cell lines. Furthermore, mitophagy signaling and paraptosis with cytoplasmic vacuolation could be blocked by antioxidant N-acetylcysteine (NAC), indicating the novel pathway was triggered by reactive oxygen species (ROS) production. An in *vivo* MDA-MB-231 xenograft tumor model revealed that CMR suppressed tumor growth by inducing vacuolation production through the same signal changes as those observed *in vitro*. These data suggest that CMR is a potential therapeutic entity for cancer treatment through a non-apoptotic pathway.

## Introduction

With the growth of aging populations and changes in modern diet, the incidence of cancer climbs yearly and is becoming more prevalent at a younger age^1^. Cancers of human reproductive system, such as breast cancer and prostate cancer, are among the most common in all cancer types, claiming more than 800,000 lives every year (WHO data). Triple negative breast cancer (TNBC), a highly invasive and metastatic phenotype with particularly poor prognosis, accounted for 10–14% in all breast cancer incidences. The lack of biomarkers (estrogen receptor, progesterone receptor and HER2), hence, the lack of effective targeted therapy, significantly limits TNBC treatment options^2^. The current standard-of-care includes the administration of poly ADP-ribose polymerase inhibitors^3^, epidermal growth factor receptor inhibitors^4^, and m-TOR inhibitors^5^, all of which have clinically significant side effects. For prostate cancer, hormone therapy, also known as androgen deprivation therapy (ADT), remains one of the primary treatment methods. When the disease progresses to an advanced stage, the cancer cells no longer need much hormone to keep growing and the disease is now categorized as castration-resistant prostate cancer (CRPC). At this point, effectiveness of ADT is greatly reduced^6^. Meanwhile, although several reports have indicated that drugs can induce apoptosis in prostate cancer cells^7^, apoptosis evasion has been observed in advanced prostate cancer and is regarded as the major obstacle in the development of effective treatment modalities^8^. Therefore, there is a pressing unmet medical need to search for a non-apoptotic cell death pathway to improve the outcomes of prostate cancer and breast cancer patients.

Forms of non-apoptotic cell death, such as mitophagy^9^, paraptosis^10^, programmed necrosis^11^ and entosis^12^, have been well-documented in literature. Mitophagy, the specific elimination of mitochondria by autophagy^9^, occurs through selective engulfment of mitochondria by autophagosomes and subsequent catabolism upon autophagosomal fusion with lysosomes^13–15^. Moreover, both defective and excessive mitophagy are linked to cell death^16^. Paraptosis is another type of non-apoptosis cell death, featuring the specific formation of cytoplasmic vacuoles^17^. Previously, it was reported that cells were characterized by a process of swelling and vacuolization beginning with the endoplasmic reticulum (ER) and mitochondria^18^, mediated by mitogen-activated protein kinases (MAPKs), and inhibited by AIP-1/Alix specifically^19^. Increasing evidence proved that paraptosis exists as intrinsic programs for cell death, morphologically distinct from apoptosis. For instance, activated microglia can trigger neuronal cell death with marked vacuolation following blockage of the caspase cascade^20^. Although the underlying molecular mechanisms for autophagy and apoptosis have been extensively characterized, the relationship between mitophagy and paraptosis is less clearly understood.

**Figure 1:**
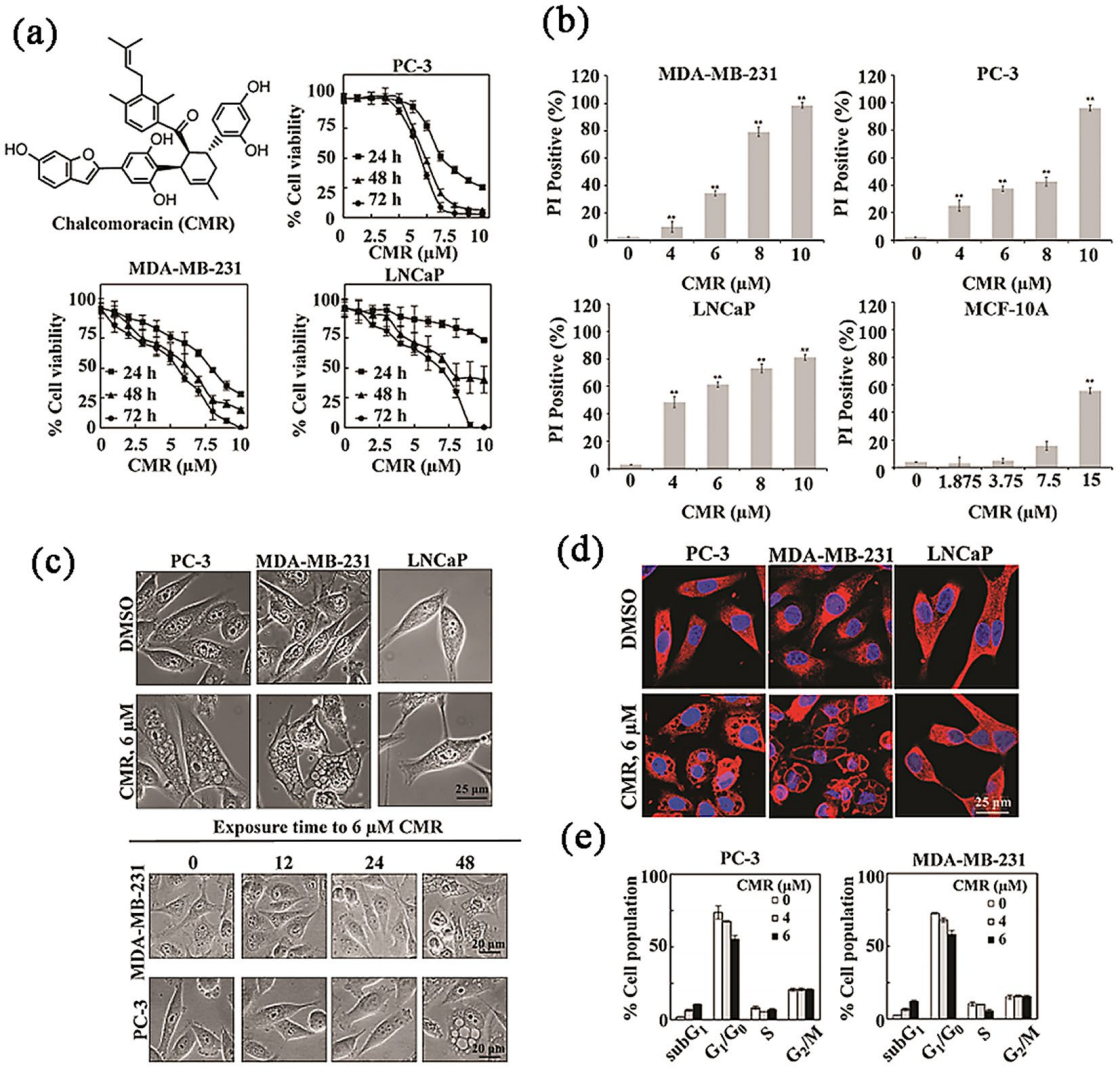
CMR inhibited cell viability, increased cell death, induced vacuolization, and blocked cell cycle in prostate and breast cancer cells. (**a**) CMR structure and dose-dependent effects on the viability of several prostate and breast cancer cell lines, PC-3, LNCaP, and MDA-MB-231, following treatment for 24–72 hours in 5% FBS-supplemented medium. Points: mean; bar: SD. Data are expressed as percentages of viable cells (treated vs. control). All assays were performed in triplicate. (**b**) CMR- induced cell death of three cancer cell lines and mammary epithelial cells MCF-10A determined by PI uptake, following treatment for 48 h. (**c**) Phase-contrast images of prostate and breast cancer cells following incubations with 6 μM CMR for 48 h (upper panel) or a range from 0–48 h (lower panel) (**d**) Immunofluorescent images of prostate and breast cancer cells following 48 h incubation with 6 μM CMR. Red: Calreticulin (1:300). (**e**) The effect of CMR on cell cycle as measured by flow cytometry. All assays were performed in triplicate.

Chalocomoracin (CMR; molecular weight: 648.69; structure: Fig. 1a) is a major secondary metabolite produced by fungus-infected mulberry leaves as a mechanism to protect the leaves via suppressing fungal germination^21^. In recent years, it has been reported that CMR has a broad spectrum of biological activities against rhinovirus, methicillin-resistant Staphylococcus aureus (MRSA)^22–24^, and human cancer cell lines^25^. There, however, has not yet been thorough investigation into the molecular mechanism of CMR in different diseases. In our study, through the investigation of CMR-induced cancer cell growth inhibition process, we observed cytoplasmic vacuoles and the decreased expression of AIP-1/Alix protein in CMR-treated human cancer cell lines PC-3 and MDA-MB-231, suggesting a non-apoptosis cell death pathway: paraptosis. To our surprise, mitophagy preceded paraptosis induced by CMR. PTEN-induced kinase 1 (PINK1), a key regulator of mitophagy, played a critical role in the whole process. To our best knowledge, this is the first report to demonstrate that CMR induced paraptosis in combination with the mitophagy pathway in cancer cell lines of human reproductive system. Thus, we believe that CMR represents a new opportunity for breast and prostate cancer treatment.

## Results

### CMR triggered non-apoptotic cell death and decreased the cell viability in MDA-MB-231 and PC-3 cells by inducing extensive cytoplasmic vacuolation

**Figure 2:**
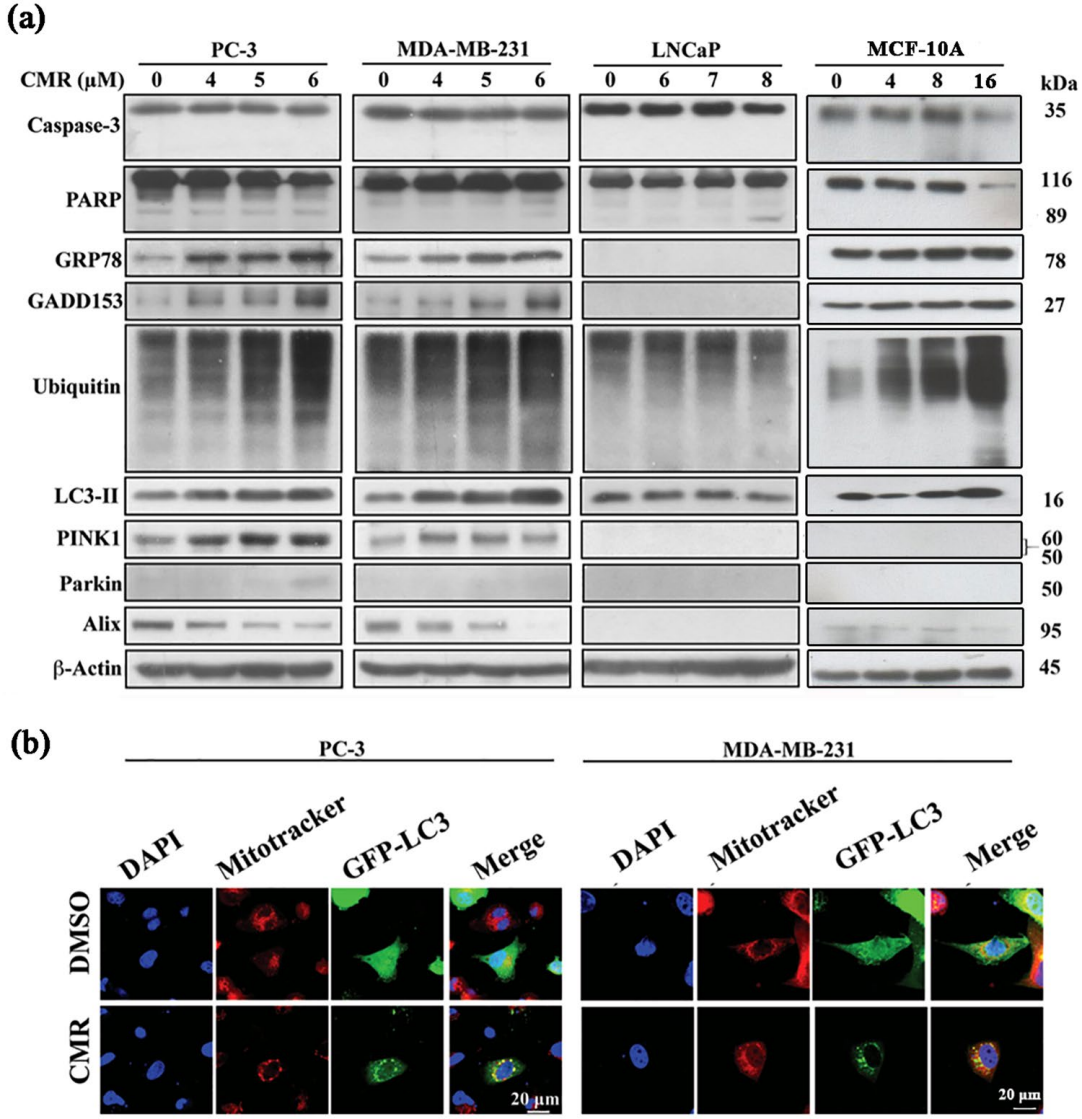
CMR induced paraptosis in PC-3 and MDA-MB-231 cell lines, but not LNCaP cell line and apoptosis in MCF-10A. (**a**) The effect of CMR on apoptosis, autophagy, ER-stress, and paraptosis-related proteins expression in PC-3, MDA-MB-231, LNCaP and MCF-10A cells. Cells were treated with varying concentrations of CMR for 48 h. Cropped blots were displayed, and original images were included in Figs S17–S20. (**b**) Immunocytochemical analysis of the effect of CMR on cellular distribution of GFP-tagged LC3 ectopically expressed in PC-3 and MDA-MB-231 cells. Cells were transfected with GFP-tagged LC3 plasmids for 24 h, treated with 6 μM CMR for 48 h, and then stained. Blue: DAPI. Red: mitotracker (0.5 μM). Green: GFP-LC3. Representative image of 3 independent experiments is shown.

We first evaluated the antiproliferative effect of CMR using PI uptake and MTT assays in three cancer cell lines, human triple-negative breast cancer line MDA-MB-231, human prostate cancer lines PC-3 and LNCaP. All three cell lines exhibited similar sensitivities to CMR with the IC_50_ values ranged from 6 µM (MDA-MB-231 and PC-3) to 8 µM (LNCaP) after 48 h of exposure to CMR (Fig. 1a and Supplementary Tables S1, S2 and S3). Furthermore, CMR (0–10 μM) induced dose-dependent and significant cell death in the three cell lines after 48 h treatment (Fig. 1b). The cytotoxicity of CMR on healthy mammary epithelial MCF-10A cells (Fig. 1b) and human normal prostate cells RWPE-2 (Fig. S1A) were much less potent than that on cancer cells. Interestingly, however, visualization of these cells post CMR exposure by phase contrast microscopy revealed differences in cell morphological changes depending on cell types. As shown in the upper panel of Fig. 1c, CMR caused extensive cytoplasmic vacuolation, where the vacuoles were clear and contained no cytoplasmic material, in PC-3 and MDA-MB-231 cells, but not LNCaP cells (Fig. 1c upper, Fig. S1B). This drug-induced vacuole formation was a late cellular event, occurring after 24 h of CMR exposure. In addition, immunofluorescent staining of endoplasmic reticulum (ER) chaperone calreticulin suggested the CMR-induced vacuoles were derived from ER in the CMR-treated PC-3 and MDA-MB-231 cells (Figs 1d and S2). This was not the case for the LNCaP cells (Figs 1d and S2). We did not find any significant accumulation in the apoptotic cell population in the sub-G1 phase when we performed cell cycle analysis using propidium iodide (Fig. 1e). Furthermore, caspase-3 activation and PARP cleavage were increased in MCF-10A, but not in PC-3 and MDA-MB-231 cells following exposure to CMR (Figs 2a and S3), confirming the cancer cells death observed was not due to apoptosis. Because cytoplasmic vacuolization in the absence of caspase activation and apoptotic marker expression is characteristic of paraptosis^20^, we hypothesized that CMR mediates paraptosis-like cell death in PC-3 and MDA-MB-231 cells.

### CMR promoted ER stress and mitophagy in MDA-MB-231and PC-3 cells

In light of the ER-associated cytoplasmic vacuolation, we investigated whether CMR affected ER stress by examining the expression of two relevant biomarkers, the 78 kDa glucose-regulated protein (GRP78) and the growth arrest- and DNA damage-inducible gene 153 (GADD153). We also measured protein ubiquitination and compared PC-3 and MDA-MB-231 cells to LNCaP cells. As shown in Figs 2a and S3, CMR increased the expression of GRP78 and GADD153 in a dose-dependent manner, as well as the accumulation of ubiquitinated protein in PC-3 and MDA-MB-231 cells. In contrast, these phenotypes were not observed in LNCaP cells. Furthermore, ER stress induction was associated with corresponding increases in LC3-II protein levels, a hallmark of autophagosome formation (Figs 2a and S3). However, in MCF-10A cells, the expressions of GRP78, GADD153 and LC3-II were softly inducted by CMR (Figs 2a and S4). We observed co-localization of LC3-II puncta with mitochondria (indicative of mitophagy) in response to CMR treatment by confocal microscopy of GFP-labelled-LC3-expressing PC-3 and MDA-MB-231 cells stained with MicroTracker Red (Fig. 2b).

### PINK1 is required for CMR-induced cytoplasmic vacuolation-interplay between mitophagy and paraptosis

CMR-provoked mitophagy and paraptosis were further investigated by analyzing the expression of two key regulators, PINK1 and ALG-2-interacting protein X (Alix), with the latter being an inhibitor of paraptosis^19,26^. While PINK1 typically undergoes rapid proteolytic degradation in the mitochondria, it is accumulated upon mitochondrial damage. This results in the recruitment of the E3 ubiquitin ligase Parkin to facilitate mitochondrial protein ubiquitination and the subsequent fusion of mitochondria to lysosomes^13,26^. As shown, CMR promoted PINK1 expression and suppressed Alix expression in a dose-dependent manner in PC-3 and MDA-MB-231 cells, while the expression levels of both regulatory proteins remained unaltered in CMR-treated LNCaP cells (Figs 2a and S3).

**Figure 3:**
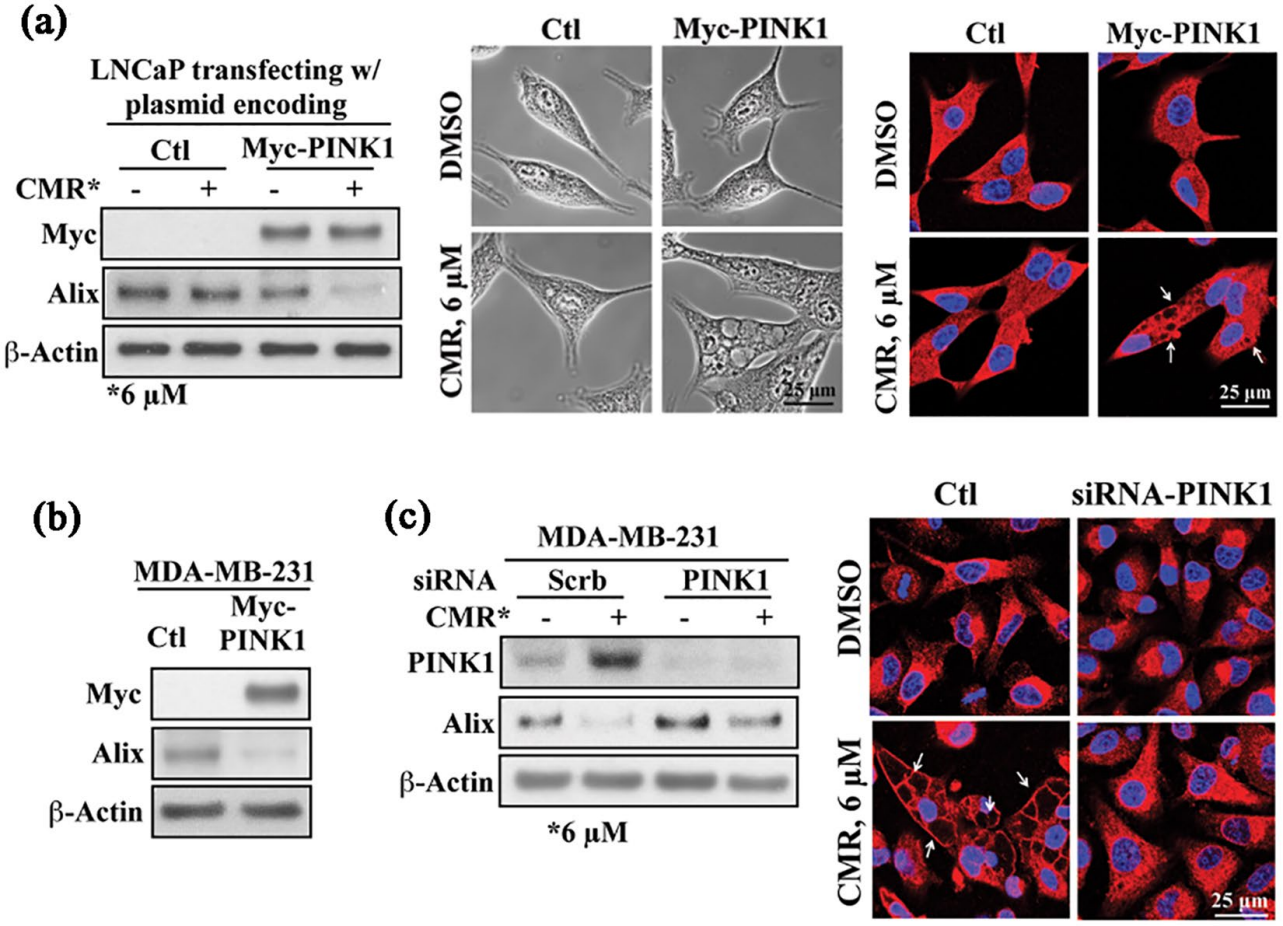
PINK1 is responsible for CMR-induced cytoplasmic vacuolation. (**a**) Analysis of the effect of ectopic expression of Myc-PINK1 on CMR-induced expression levels of Alix and cytoplasmic vacuolation in LNCaP cells by Western blot (left panel), phase-contrast (middle panel) and immunocytochemistry (right panel). (**b**) The effect of ectopic expression of Myc-PINK1 on the expression levels of Alix in MDA-MB-231 cells as measured by Western blot. (**c**) The effect of siRNA-mediated knockdown of PINK1 on CMR-induced changes in the expression of Alix in MDA-MB-231 cells as analyzed by Western blot (left panel) and immunocytochemistry (right panel). Red: Calreticulin. All cells were treated with 6 μM CMR for 48 h. Cropped blots were displayed, and original images were included in Figs S21–S23.

Based on the findings above, we propose that PINK1 plays an integral role in CMR-mediated paraptosis, which was further corroborated by the following two lines of evidence. First, ectopic expression of Myc-PINK1 rendered LNCaP cells susceptible to CMR induction of paraptosis. As demonstrated in Figs 3a and S5, exposure of PINK1-expressing LNCaP cells to CMR resulted in reduced expression of Alix (Fig. 3a, left panel, Supplementary Fig. S5) and cytoplasmic vacuolation (Fig. 3a, center panel) as visualized by phase contrast microscopy and immunofluorescent staining of calreticulin (Fig. 3a, right panel, Supplementary Fig. S6A). Secondly, PINK1 also suppressed the expression of Alix in MDA-MB-231 cells (Figs 3b and S6B), suggesting the association between CMR-mediated downregulation of Alix expression and upregulation of PINK1 expression. Moreover, PINK1 depletion via siRNA protected MDA-MB-231 cells from CMR-induced changes in Alix expression (Fig. 3c, left panel, Supplementary Fig. S5C) and cytoplasmic vacuolation (Fig. 3c, right panel, Supplementary Fig. S6B).

### CMR-induced cytoplasmic vacuolation requires active protein synthesis

**Figure 4:**
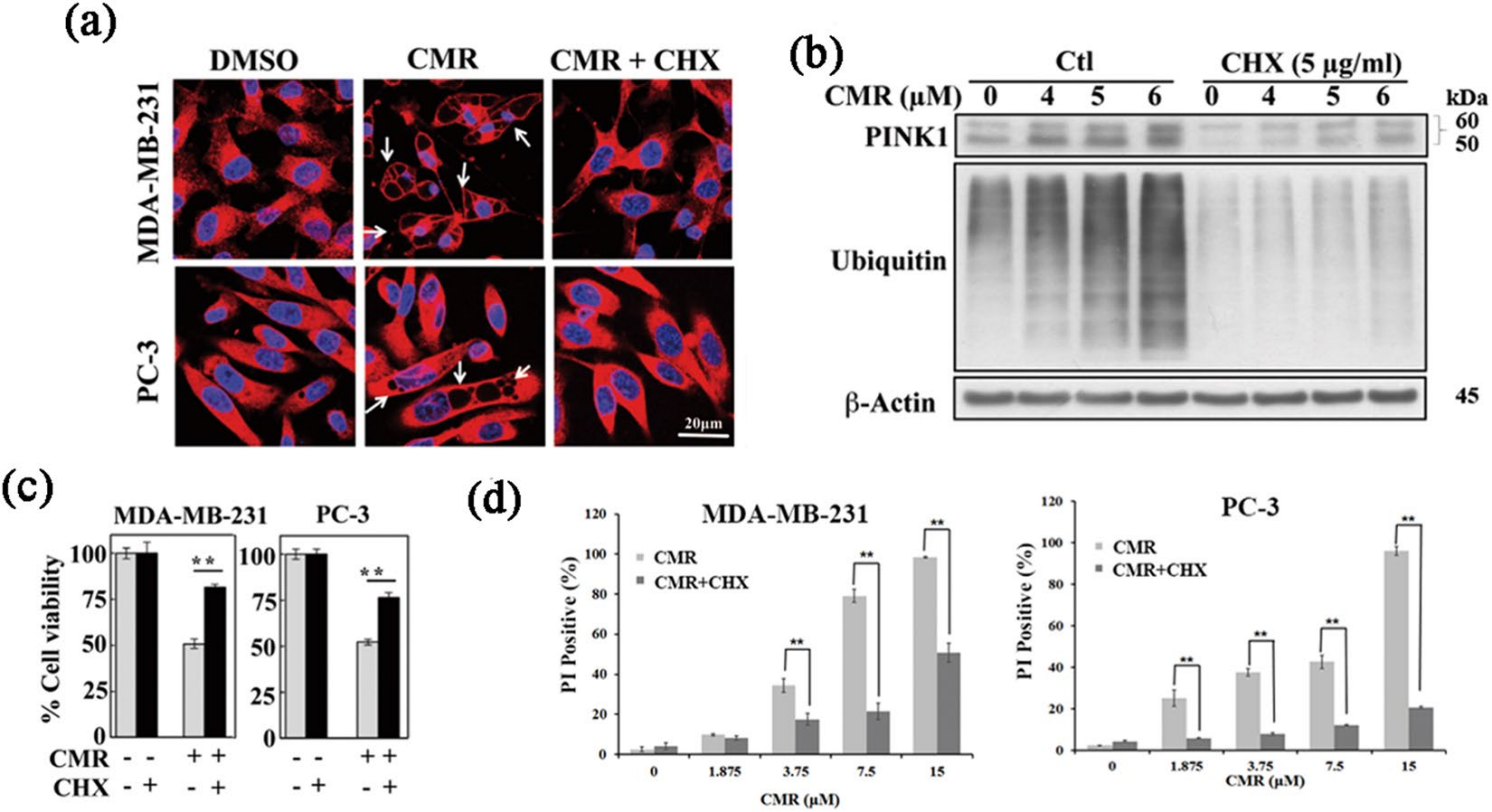
Active protein synthesis is required for CMR-induced cytoplasmic vacuolation. Analysis of CMR-induced cytoplasmic vacuolation in MDA-MB-231 and PC-3 cells by (**a**) immunocytochemistry and (**b**) Western blot analysis. Cropped blots were displayed, and original images were included in Fig. S24. The effect of cycloheximide (CHX) on (**c**) cell viability and (**d**) cell death was detected by MTT and PI uptake assays. Cells were pre-treated with 5 μg/ml CHX for 1 h. All cells were treated with 6 μM CMR for 48 h. Red: Calreticulin.

The causal relationship between CMR and the induction of mitophagy and paraptosis was further supported by the ability of cycloheximide (CHX), a general inhibitor of protein synthesis, to block CMR-induced cytoplasmic vacuolation (Figs 4a and S7A). As a reporter of mitochondrial damage, PINK1 is typically maintained at very low levels to prevent mitophagy of healthy mitochondria^14^. However, in response to mitochondrial damage, protein synthesis of full-length PINK1 is ramped up, leading to the rapid accumulation and activation of Parkin, which can be blocked by CHX^27,28^. Consistent with the requirement of protein synthesis for mitophagy initiation, pretreatment of cells with CHX abrogated the ability of CMR (6 µM) to induce PINK1 upregulation and protein ubiquitination (Figs 4b and S7B), protecting MDA-MB-231 and PC-3 cell viability (Fig. 4c) against CMR-induced cell death (Fig. 4d).

### ROS-mediated dysregulation of mitochondrial membrane potential and Ca^2+^ homeostasis contributes to CMR-induced paraptotic death

**Figure 5:**
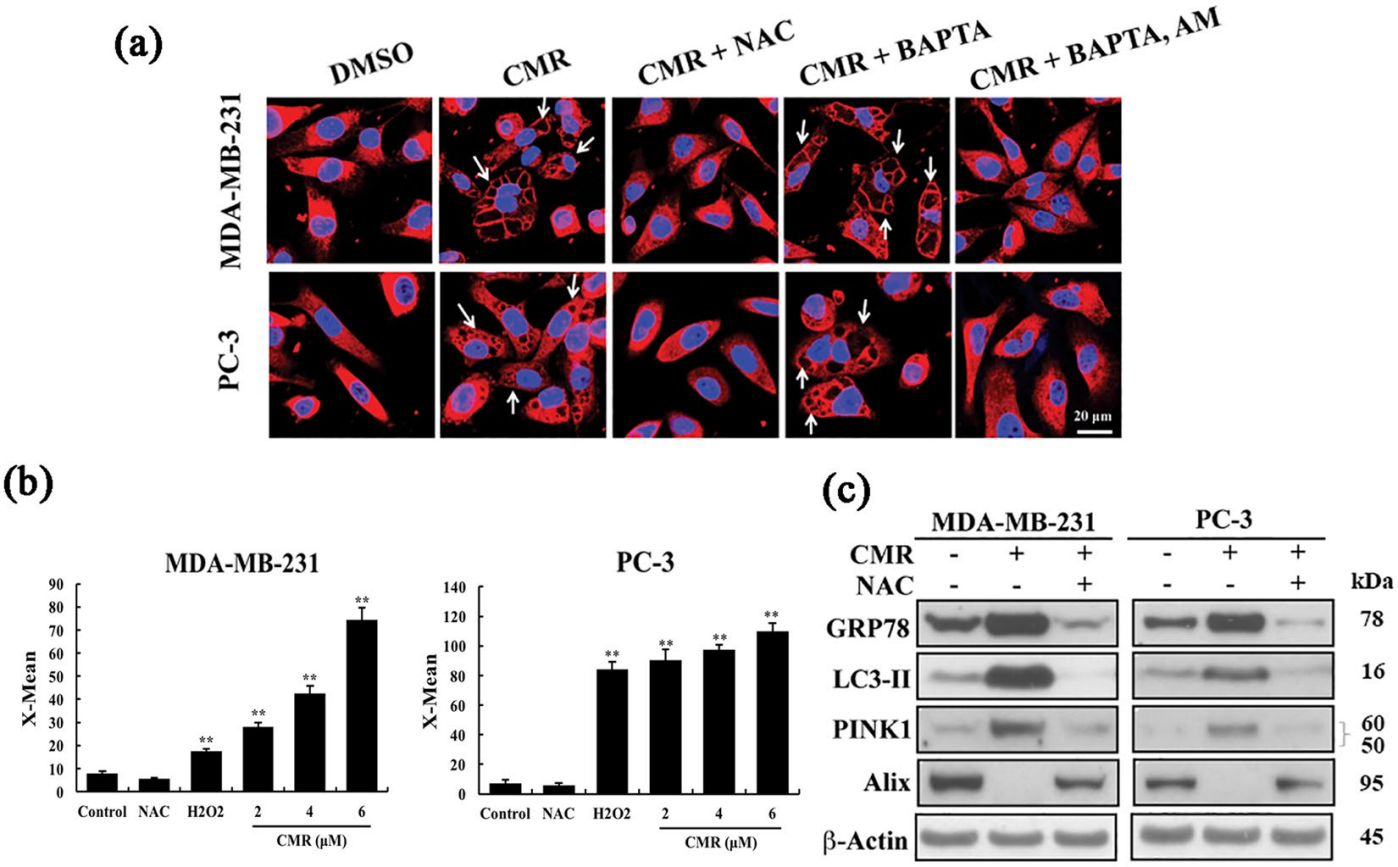
Oxidative stress and intracellular calcium plays important roles in CMR-induced cytoplasmic vacuolation. (**a**) The effect of NAC (5 mM), BAPTA (2.5 μM), and BAPTA-AM (2.5 μM) on CMR-induced cytoplasmic vacuolation in MDA-MB-231 and PC-3 cells as measured by immunocytochemistry. Cells were pre-treated with NAC for 1 h, BAPTA and BAPTA-AM for 2 h. (**b**) Induction of ROS in MDA-MB-231 and PC-3 cells by CMR, where H_2_O_2_ (100 μM) and NAC were used as positive and negative control. Points: mean; bar: SD. *p < 0.05 and **p < 0.01vs. control. (**c**)The effect of NAC on CMR-induced oxidative stress, mitophagy, and paraptosis in MDA-MB-231 and PC-3 cells as analyzed by Western blot. Cropped blots were displayed, and original images were included in Figs S25 and S26. All cells were treated with 6 μM CMR for 48 h.

Importantly, CMR-facilitated cytoplasmic vacuolation could be blocked with the antioxidant N-acetylcysteine (NAC) and the intracellular Ca^2+^ chelator BAPTA-AM, indicating that oxidative stress and dysregulated Ca^2+^ homeostasis played a role in mediating this drug-induced paraptosis (Figs 5a and S8). The involvement of oxidative stress was further corroborated by the CMR dose-dependent induction of ROS production in both MDA-MB-231 and PC-3 cells (Figs 5b and S9). In addition, NAC abolished CMR-induced changes in biomarkers of oxidative stress (i.e., GRP78), mitophagy (i.e., LC3-II and PINK1), and paraptosis (i.e., Alix) (Figs 5c and S10).

**Figure 6:**
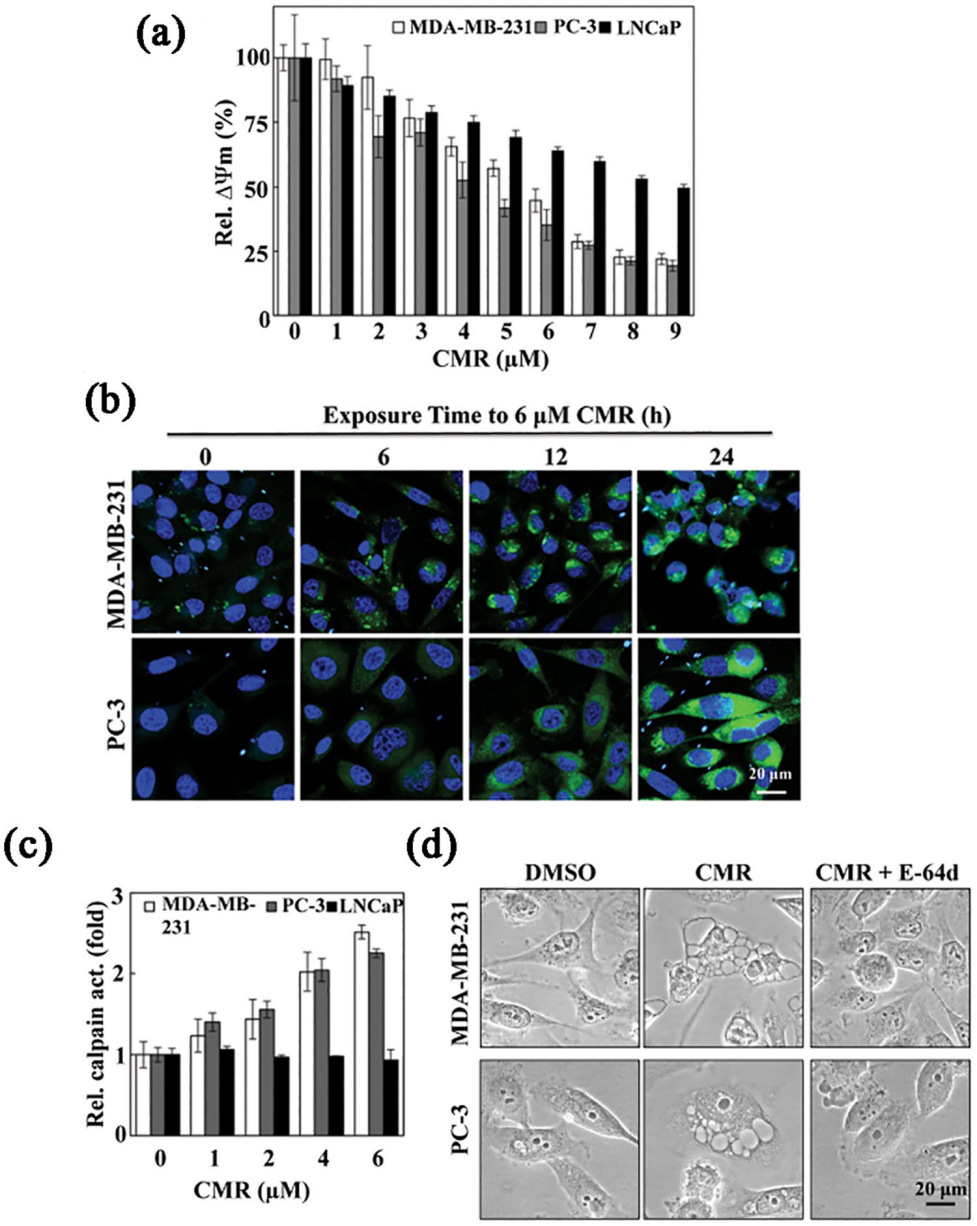
CMR-induced paraptotic death is the result of ROS-mediated loss of mitochondrial membrane potential and Ca^2+^ homeostasis. (**a**) The effect of CMR on the mitochondrial membrane potential in MDA-MB-231, PC-3, and LNCaP cells. (**b**) The effect of CMR on the increase of cytoplasmic calcium concentrations in a time-dependent manner in MDA-MB-231 and PC-3 cells as measured by immunocytochemistry, where Fura-2/AM (4  μM) was used as an indicator of cytoplasmic calcium. (**c**) The effect of CMR on the increase of calpain activity in a dose-dependent manner in MDA-MB-231 and PC-3 cells. (**d**) The effect of E-64d (10 μM), a calpain inhibitor, on CMR-induced cytoplasmic vacuolation in MDA-MB-231 and PC-3 cells as visualized by phase contrast. Cells were pre-treated with 10 μM E-64d for 1 h. All cells were treated with 6 μM CMR for 48 h.

Because mitochondria are major sites of ROS generation, we hypothesized that ROS production in this system would result in the loss of mitochondrial membrane potential (ΔΨm), which has been found to activate the PINK1-Parkin signaling pathway and, consequently, trigger mitophagy^27–29^. As shown in Fig. 6a, CMR treatment of MDA-MB-231 and PC-3 cells lead to the loss of ΔΨm in a dose-dependent manner in contrast to the maintenance of ΔΨm in LNCaP cells under the same conditions.

The loss of ΔΨm has been known to be associated with increased intracellular free Ca^2+^ concentrations^30^. Therefore, we examined the effect of CMR treatment on intracellular Ca^2+^ levels using Fura-2, a ratiometric fluorescent dye used for calcium imaging. As shown, exposure of MDA-MB-231 and PC-3 cells to 6 µM CMR triggered a time-dependent increase in intracellular Ca^2+^ concentrations (Figs 6b and S11), which appeared to precede cytoplasmic vacuole formation in both cell lines (Fig. 1b, lower panel).

Pursuant to this finding, we turned our attention to calpains, cytoplasmic Ca^2+^-activated proteases, due to their roles in caspase-independent cell death^31^. We found that CMR treatment led to an elevation in intracellular Ca^2+^ that was accompanied by increased calpain activity in MDA-MB-231 and PC-3 cells, but not in LNCaP cells (Fig. 6c). Moreover, inhibition of calpains by the thiol protease inhibitor E-64d blocked CMR induction of cytoplasmic vacuolation in these two cell lines (Fig. 6d) via a mechanism that currently remains to be delineated.

### The MAP kinase is involved in CMR-induced paraptosis

**Figure 7:**
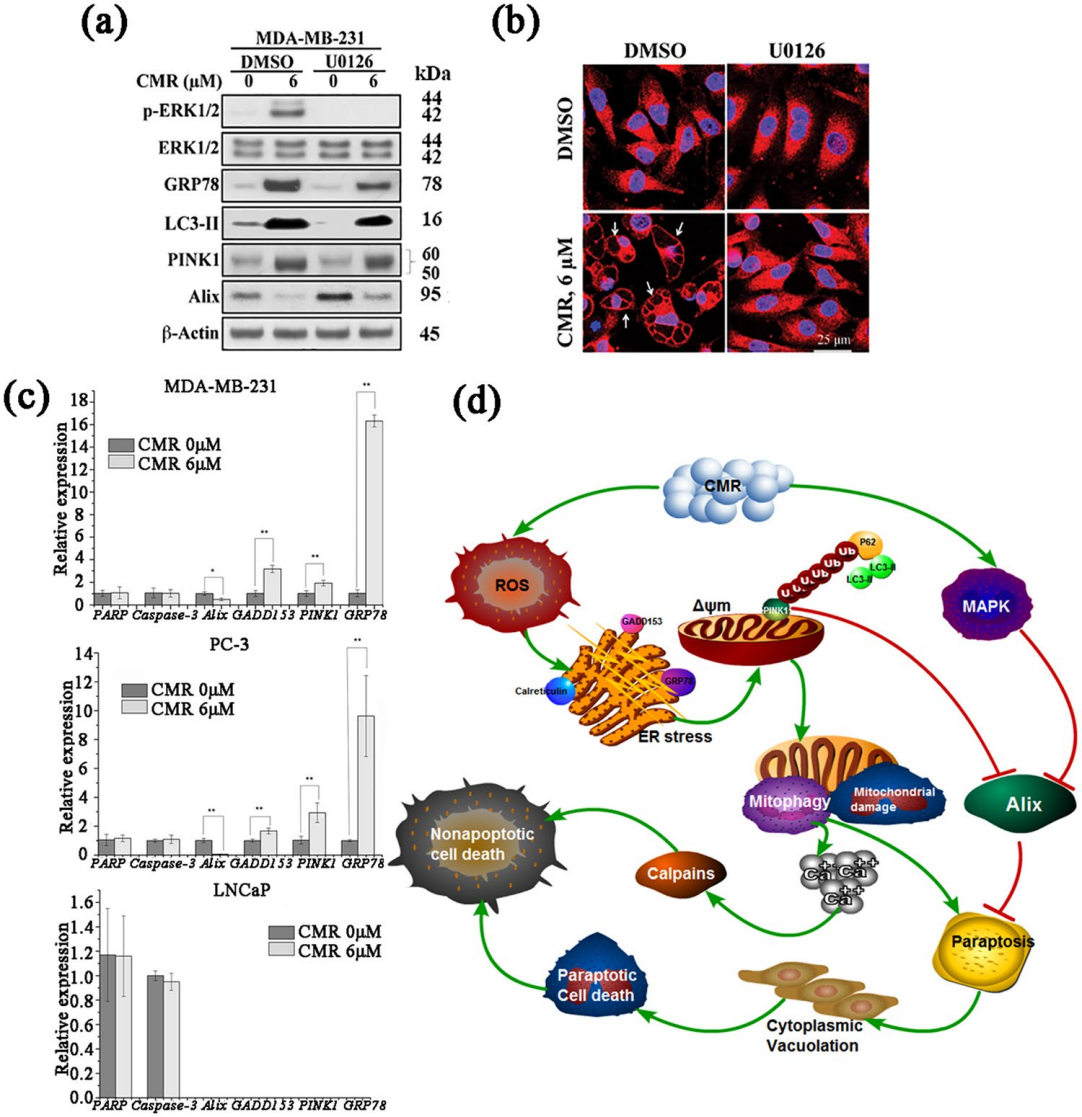
MAP kinase plays a role in CMR-induced paraptosis, and CMR regulated genes of paraptosis and mitophagy. (**a**,**b**) The effect of U0126 (10 μM) on CMR-induced paraptosis in MDA-MB-231 was measured by (**a**) Western blot and (**b**) immunocytochemistry. Cropped blots were displayed, and original images were included in Figs S27 and S28. Cells were pre-treated with 10 μM U0126 for 1 h. (**c**) Q-PCR analysis was based on the method. Points: mean; bar: SD. *p < 0.05 and **p < 0.01vs. control. (**d**) Model of the mechanism by which CMR induces paraptosis of cancer cells through mitophagy and activation of ERK signaling. All cells were treated with 6 μM CMR for 48 h.

Another important signaling mediator in the regulation of paraptosis, in addition to Alix, is MAP kinase^19^. As presented in Figs 7a and S12A, exposure of MDA-MB-231 cells to CMR led to increased ERK1/2 phosphorylation, which is indicative of MAPK activation. Pharmacological inhibition of MAPKs by U0126 led to increased Alix levels, suggesting a role for MAPKs in regulating Alix expression. When cells were simultaneously treated with CMR and U0126, the suppressive effect of CMR on Alix expression was abrogated, thereby maintaining Alix expression at basal levels. As a consequence, U0126 also inhibited CMR-induced cytoplasmic vacuolation (Figs 7b and S12B). Notably, U0126 had no appreciable effect on the ability of CMR to induce oxidative stress, LC3-II conversion, or PINK1 upregulation, suggesting that these cellular responses are distinct from MAP kinase activation.

### CMR regulated the expression of paraptosis and mitophagy genes

Quantitative real-time PCR (Q-PCR) was used to further investigate the anti-tumor mechanism of CMR *in vitro*. Our results showed that the expressions of *PINK1*, *GADD153*, *GRP78* genes increased, and *Alix* gene decreased after CMR treatment. Consistent with the protein expression, *Caspase-3* and *PARP* genes were not affected by CMR (Fig. 7c). In summary, CMR functions at the protein level as well as their mRNA levels.

### CMR inhibits tumor growth *in vivo*

**Figure 8:**
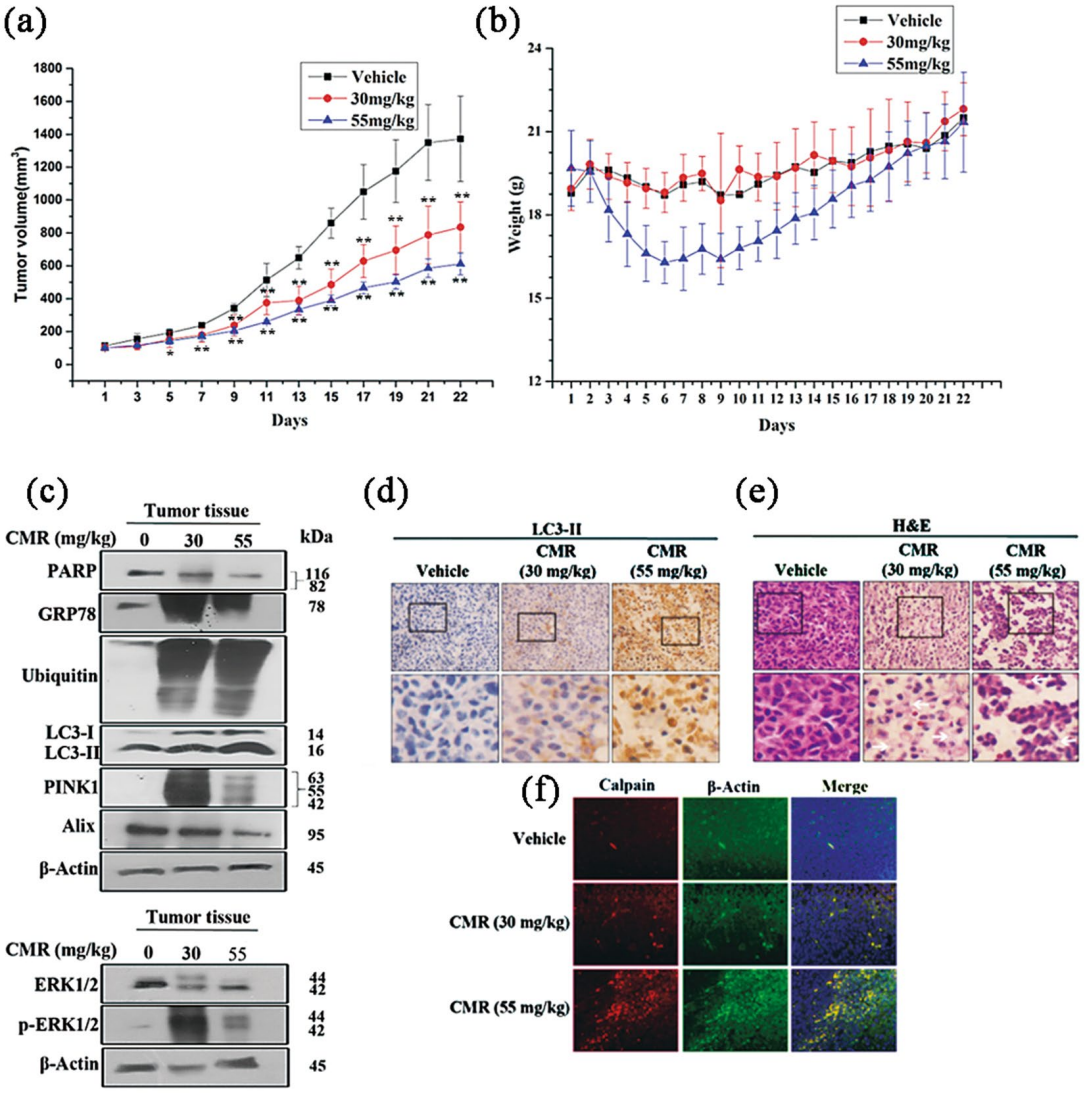
CMR inhibits MDA-MB-231 tumor growth *in vivo* and causes the same biochemical signals changes as *in vitro*. (**a**–**c**) The effect of CMR treatment on tumor growth in mice as measured by (**a**) tumor volume, (**b**) body weight, and (**c**) protein expression levels via Western blot. The treatment period was 21 days. Cropped blots were displayed, and original images were included in Figs S29 and S30. (**d**) Immunohistochemistry of LC3-II (upper panel: 100x; lower panel: magnification of the boxed areas in the vehicle- and CMR-treated groups, 400x) of tumor tissue. The quantity and color depth of brown granules correlated with LC3-II content in tumor tissues. (**e**) Tumor sections were H&E stained (upper panel: 100x; lower panel: magnification of the boxed areas in the vehicle- and CMR-treated groups, 400x) to visualize vacuolation (arrow). (**f**) Immunofluorescent images of tumor sections after CMR treatment. Paraffin embedded tumor xenograft was incubated with anti-calpain 1 and anti-β-Actin antibodies to quantify the concentration of Ca^2+^ in tumor tissues. Blue: DAPI. Red: Calpain1 (1:200). Green: β-Actin (1:200).

To assess the effect of CMR on human tumors engrafted into nude mice, we conducted an *in vivo* study using carefully-selected CMR doses. Treatment with 30 mg/kg and 55 mg/kg CMR resulted in tumor growth inhibition of 46% and 54%, respectively, compared to the vehicle control, and no significant weight loss was observed in any mice (Fig. 8a,b). We observed via Western blot that CMR treatment led to an upregulation of PINK1 and LC3-II in the tumor samples, indicative of mitophagy induction. Furthermore, Alix was downregulated and ERK was activated, indicating the occurrence of paraptosis (Fig. 8c, Supplementary Figs S13 and S14A). Additionally, CMR induced upregulation of GRP78 and protein ubiquitination *in vivo* (Fig. 8c, Supplementary Figs S13 and S14A). In contrast, CMR treatment had no effect on PARP and caspase-3 (Figs 8c and S14A)

### *In vivo* vacuolation and LC3-II and calpain 1 accumulation

When compared to the vehicle, the population of LC3-II-positive cells increased following treatment with CMR (Figs 8d and S15A), consistent with vacuolation observed in tumor tissues using hematoxylin and eosin (H&E) staining (Figs 8e, S15B). Furthermore, we detected calpain 1 expression, which correlated with Ca^2+^ concentrations in tumor tissues. Calpain 1 protein levels increased in a dose-dependent manner in response to CMR (Figs 8f and S15C). Overall, these results suggest that CMR induced MDA-MB-231 cell death by enhancing the frequency of mitophagy and vacuolation *in vivo*, in line with the aforementioned *in vitro* findings.

## Discussion

Previous reports indicated that human cancer cell lines, such as A549, Be17402, BGC823, HCT-8 and A2780, can be inhibited by CMR significantly^21,32^. However, further exploration of CMR on antitumor mechanisms are less clearly understood. MDA-MB-231 cells (androgen receptor positive) and PC-3 cells (androgen-independent) mainly were considered in the research because of the poor therapeutic effect and prognosis of TNBC and CRPC^33^. Recently, it has reported that androgen receptor may be the therapeutic target of androgen-driven triple-negative breast cancer patients, and anti-androgens, widely used to treat metastatic CRPC, can be used for TNBC treatment^34^. In other words, common targets exist in TNBC and CRPC, but few studies have been done. Therefore, it is significant and necessary to explore a pathway for the treatment of TNBC and CRPC. Our study has shown that CMR can lead to cell death in breast cancer cell line MDA-MB-231, prostate cancer cell lines PC-3 and LNCaP, but the anti LNCaP effect was weaker than the other two cell lines. Here, we found that MDA-MB-231 and androgen-independent PC-3 cells could response to the CMR-induced cytoplasmic vacuoles, but androgen-responsive LNCaP cells couldn’t. This process began with ROS production and MAPK activation, indicative of paraptosis, a non-apoptotic pathway. The main features of paraptosis include extensive cytoplasmic vacuolation, the absence of significant cell membrane blebbing and nuclear shrinkage, or pyknosis^35^. In contrast to cytoplasmic vacuolation death^36^, another type of non-apoptosis cell death-, paraptosis is associated with mitochondrial swelling^14^. The protein AIP1/Alix was identified independently by two groups as a protein interacting with the cell death-related calcium-binding protein ALG-2, inhibiting paraptosis^19^. Although much less is known about the biochemical mediators of this type of cytoplasmic cell death, investigating the paraptosis pathway holds great value for cancer therapy as an alternative to apoptosis. During our exploration into the mechanism by which CMR suppresses cancer cell proliferation, we noted the unique ability of CMR to promote paraptosis in conjunction with extensive cytoplasmic vacuolation in PC-3 prostate and MDA-MB-231 breast cancer cells. CMR inhibited the expression of AIP1/Alix protein in the two cell lines, suggesting an activation of paraptosis.

Another feature of paraptosis is caspase-independent^37,38^. Our data revealed that caspase-3 activation and PARP cleavage were not altered in PC-3 and MDA-MB-231 cells following exposure to CMR, and no significant accumulation in the apoptotic cell population in the sub-G1 phase was noted (Fig. 1d). Upon further exploration of the induction mechanism of paraptosis in the CMR-treated cells, exposure of MDA-MB-231 cells to CMR led to increased ERK1/2 phosphorylation, indicative of MAP kinase activation, which is related to paraptosis as reported^19^. Even more remarkably, we found another pathway to mediate CMR-induced paraptosis. Evidence suggests this CMR-induced paraptosis was preceded by mitophagy. The specific autophagic elimination of mitochondria is the selective engulfment of mitochondria by autophagosomes and their subsequent catabolism by lysosomes^13^. We found that GFP-LC3B protein, a marker of autophagosome formation, translated onto the mitochondrial surface along with full-length PINK1 accumulation, a molecular sensor of damaged mitochondria^3^. LNCaP cells only poorly express PINK1 and are not sensitive to CMR-mediated Alix downregulation. Based on these findings, we found that CMR can regulate mitophagy and paraptosis genes in MDA-MB-231 and PC-3 cells. However, in LNCaP cells, expressions of *GADD153*, *GRP78* and *Alix* genes could not be detected (Fig. 7c). Therefore, it can be inferred that CMR regulated protein expression by influencing genes ultimately leading to mitophagic and paraptotic cell death. Precisely because LNCaP cells lack the expression of related genes, it showed the difference from that of CRPC PC-3 Cells. Furthermore, we ectopically expressed Myc-PINK1 in LNCaP cells, and found Myc-PINK1 rendered LNCaP cells susceptible to the paraptotic effects of CMR. In addition, siRNA-mediated knockdown of PINK1 protected MDA-MB-231 cells from the Alix downregulation by CMR. Altogether this shows PINK1 plays an important role in mitophagy and paraptosis.

In addition, The PINK1-Parkin pathway is important in regulating clearance of dysfunctional mitochondria via mitophagy^39^. The accumulation of PINK1 on the mitochondrial surface induced translocation of Parkin from the cytosol to damaged mitochondria, and then the recruited Parkin promoted the degradation of mitochondria through mitophagy^40^. In our study, CMR upregulated PINK1 expression and activated Parkin, leading to cell ubiquitination (Fig. 2a). Because of the higher division rate of the cancer cells, more ubiquitinated proteins were accumulated^41^ and more damages were caused compared with normal cells after CMR treatment (Fig. 1b). Based on these findings, CMR-induced mitophagy pathway may be a novel strategy for breast and prostate cancer treatment.

Mitophagy is a process that selectively degrades mitochondria, the main site of continuous ROS production in most mammalian cells^42^. Recent studies have demonstrated that ROS overproduction elevates Ca^2+^ concentrations to abnormal levels^31,43,44^. A substantial rise in ROS and massive Ca^2+^ release is associated with the opening of the mitochondrial permeability transition pore (mPTP), which eventually leads to cell death^44–46^. We investigated the cause of mitophagy after the CMR treatment by conducting detection assays based on mitochondria in PC-3 and MDA-MB-231 cell lines. Our data showed that CMR induced oxidative stress, followed by intracellular Ca^2+^ release and increased activity of the calcium-dependent, non-lysosomal cysteine protease family protein calpain. This resulted in dysregulation of mitochondrial membrane potential, which led to increased PINK1 expression. Through immunofluorescent staining with endoplasmic reticulum (ER) chaperone calreticulin and GRP78 and GADD153 expression caused by the unfolded protein response (UPR)^47^ in the CMR-treated PC-3 and MDA-MB-231 cells to visualize vacuole localization, we also found that CMR-mediated cytoplasmic vacuolation is related to ER-stress in the process of ROS-induced mitophagy (Figs 1d and S1).

Moreover, CMR suppressed xenograft tumor growth *in vivo* via the same mechanism delineated *in vitro*. Importantly, CMR was relatively less toxic in nude mice, normal prostate cells (RWPE-2) and epithelial breast cells (MCF-10A) without mitophagy and paraptosis induced. Therefore, in the treatment of cancer, select the appropriate dose can make no toxic effects in normal cells. The CMR-induced mitophagy and paraptosis pathway is outlined in Fig. 7d. Overall, CMR was found to have antitumor properties through the mediation of a unique and delicate interplay between mitophagy and paraptosis *in vivo* and *in vitro*. highlighting the translational potential of this natural compound to serve as a chemopreventive agent. Further investigation of this compound will guide practical applications for CMR.

## Materials and Methods

### Cell lines and Cell Culture

All cancer cell lines were purchased from the American Type Culture Collection. Media used for the maintenance of these cell lines are listed as follows: MDA-MB-231, Dulbecco’s Modified Eagle Medium (DMEM, Life Technologies, 12430-054); LNCaP and PC-3, Roswell Park Memorial Institute (RPMI) 1640 medium (RPMI 1640, Life Technologies, 22400-089); All media was supplemented with 10% fetal bovine serum (FBS, Gibco, 16000-044) and penicillin-streptomycin unless otherwise indicated. Mammary epithelial cells MCF-10A were also supplied by the American Type Culture Collection. Mammary Epithelial Cell Medium (MEpiCM, Sciencell, 7611) was used for incubation, consisting of 500 ml of basal medium, 5 ml of mammary epithelial cell growth supplement (MEpiCGS, Sciencell, 7652) and 5 ml of penicillin/streptomycin solution (P/S, Sciencell, 0503). Cells were incubated at 37 °C in 5% CO_2_ in a humidified incubator.

### Antibodies, Fluorescent dyes and Chemicals

Mouse monoclonal antibodies were used for Parkin, Alix, and Thr(P)^202^/Tyr(P)^204^-Erk1/2 (Cell Signaling Technology, 4211, 2174 and 14227), Caspase-3 and Ubiquitin (Cell Signaling Technology, 9668 and 3936). Rabbit antibodies were used for β-actin, PARP, LC-3, and Erk1/2 (Cell Signaling Technology, 8457, 9542, 3868, and 4696), GRP78 and GADD153 (Santa Cruz, sc-376768 and sc-7351), Calreticulin (Abcam, ab108395), PINK1 (Novus Biologicals, NBP2-36488). Goat antibodies were used for calpain, calpain 1, calpain 2 (Santa Cruz, sc-2448, sc-7530 and sc-7532), IgG-horseradish peroxidase (HRP), and goat anti-mouse IgG-HRP (ImmunoResearch Laboratories) and FITC goat anti-rabbit IgG (Beyotime, A0562). Donkey antibodies were used for Alexa Fluor 555 donkey anti-rabbit IgG (Life Technologies, bs-0295D-AF555) and Cy3 donkey anti-goat IgG (Beyotime, A0502). Fluorescent dyes included MitoTracker® Deep Red FM (Life Technologies, M22426), and Fura-2/AM (Abcam, ab120873). For the chemicals used in this study, Chalcomoracin (CMR) was isolated by Jingkui Tian’s lab^48^, the Key Laboratory of Biomedical Engineering, Zhejiang University. Other chemicals used included cycloheximide (CHX, Biosharp, bs168a), *N*-acetylcysteine (NAC, Sigma-Aldrich, A9165-25), BAPTA, BAPTA-Acetoxymethyl ester, Dantrolene, Ruthenium Red, 2-Aminoethoxydiphenyl borate (2-APB) and E-64d (Cayman Chemical, 11706, 15551, 11103-72-3, 17146 and 13533), and U0126 (ThermoFisher, PHZ1283). Immunohistochemistry, immunofluorescence, and H&E staining of tumor tissue used hematoxylin (C0107), eosin (C0109), DAPI (C1002), sodium citrate buffer (M019) and BSA (ST023) from Gefan; DAB from Beyotime, P0202; Goat serum from Gibco, 16210064; and methanol, ethanol, xylene, hydrogeno peroxide and formaldehyde solution from Sinopharm. Primers: PARP (Forward primer: CGGAGTCTTCGGATAAGCTCT; Reverse primer: TTTCCATCAAACATGGGCGAC), Caspase-3 (Forward primer: CATGGAAGCGAATCAATGGACT; Reverse primer: CTGTACCAGACCGAGATGTCA), Alix (Forward primer: ATGGCGACATTCATCTCGGTG; Reverse primer: CGCTTGGGTAAGTCTGCTGG), GADD153 (Forward primer: GGAAACAGAGTGGTCATTCCC; Reverse primer: CTGCTTGAGCCGTTCATTCTC), GRP78 (Forward primer: CATCACGCCGTCCTATGTCG; Reverse primer: CGTCAAAGACCGTGTTCTCG), PINK1 (Forward primer: GCCTCATCGAGGAAAAACAGG; Reverse primer: GTCTCGCCAACGGGTC).

### Cell Viability Assays

The effect of CMR or CHX pre-treatment on cell viability was assessed using the 3-(4,5-dimethylthiazol-2-yl)-2,5-diphenyl-2H-tetrazolium bromide (MTT, Solarbio, M8180) assay. Cells were seeded into 96-well plates in their respective medium supplemented with 10% FBS and incubated for 24 h before exposed to a range of concentrations of test articles dissolved in DMSO in 5% FBS-supplemented medium for 24–72 h (CMR treatment) or 48 h (CHX pre-treatment). Subsequently, the medium was removed and replaced with 200 μl of 0.5 mg/ml MTT in 10% FBS-containing medium, and the cells were incubated in the CO_2_ incubator at 37 °C for 1 h. Supernatants were removed from the wells, and the MTT dye was solubilized in 200 μL/well DMSO (Sigma, 67-68-5). Absorbance was measured at 570 nm on a plate reader. Each condition was tested with 6 replicates and all assays were performed in triplicate.

### Flow cytometry for cell death assay

Cells were collected by trypsinization and subsequent centrifugation, resuspended in ice-cold PBS containing 0.7 µg/ml of propidium iodide (Sigma, #P4170) and analyzed using a FACSCalibur (BD Biosciences) flow cytometer and FlowJo analysis software (Tree Star, Inc.). All assays were performed in triplicate.

### Transient Transfection

Cells were transfected with the indicated plasmids or siRNA using Lipofectamine® 2000 transfection reagent (Life Technologies, 11668-019) according to the manufacturer’s instructions. Briefly, 3 μg of plasmid DNA or siRNA was added to each well with 6 μL Lipofectamine 2000. Treatments were initiated 48 h after 24 h transfection. The plasmids encoding Myc-PINK1 and GFP-LC3 were obtained from Addgene. The siRNA-PINK1 was purchased from OriGene. Expression of each plasmid was confirmed by immunoblotting.

### Immunoblotting

Drug-treated cells were collected from 6 cm dishes by scraping and centrifugation. The cells were washed once with phosphate-buffered saline (PBS), and then lysed in a lysis buffer containing 1% sodium dodecyl sulfate (SDS), 10 mM ethylenediaminetetraacetic acid (EDTA), 50 mM Tris-HCl (pH 8.1) and (Sigma-Aldrich, P8340). Lysates were sonicated for 15 s to shear genomic DNA and then centrifuged at 13,000 × *g* for 10 min. Concentrations of proteins in the supernatants were quantified using the Micro BCA Protein Assay Kit (Pierce Biotechnology, 23235). Equal amounts of each protein were resolved in a SDS-polyacrylamide gel and transferred to a polyvinylidene fluoride (PVDF, Bio-Rad, 1620184) membrane. The transblotted membrane was blocked with Tris-buffered saline containing 0.1% Tween-20 (TBST) and 5% non-fat milk for 30 min, and then incubated with the appropriate primary antibody (1:500-1: 4,000 dilution) in TBST at 4 °C overnight. The membrane was washed three times with TBST for a total of 30 min, incubated with goat anti-rabbit or anti-mouse IgG-HRP conjugates (1: 5,000 dilution) for 2 h at room temperature, and then washed again as described previously. Western Lighting Chemiluminescence Reagent Plus (Perkin-Elmer, NEL103001EA) was used to develop the images and the immunoblots were visualized by enhanced chemiluminescence.

### Immunofluorescent and Phase Contrast Imaging

Cells that had been treated with drug or transfected with plasmid were washed with cold PBS, fixed with 4% formaldehyde in PBS for 20 min at 37 °C, permeabilized with 0.5% Triton X-100 in PBS for 10 min at room temperature, and then blocked with 1% BSA in PBS for 10 min. After washing with PBS, the cells were incubated overnight with anti-Calreticulin antibody in 1% BSA at 4 °C. The cells were then incubated with Alexa Fluor 555 donkey anti-rabbit IgG in 1% BSA for 2 h at room temperature. Nuclei were stained with DAPI in Vectashield mounting medium (Vector Laboratories). Confocal images were obtained using the 63X oil immersion lens in an Olympus FV1000 confocal microscope (Olympus Corp.), while phase contrast images were obtained with the 63X oil immersion lens in an Olympus SZH Zoom Stereo Microscope (Olympus Corp.).

### Flow Cytometry for cell cycle

Drug-treated cells were harvested in ice-cold PBS (1 × 10^6^ cells/200 μl) and then fixed in 500 μl 100% methanol. For cell cycle analysis, the fixed cells were resuspended in 500 μl propidium iodide (PI) staining solution (80 μg/ml PI, 100 μg/ml RNase, and 0.1%, v/v, Triton X-100 in PBS) and incubated at 37 °C for 30 min in the dark. The populations of cells within each cell cycle phases were evaluated using a FACSCalibur (BD Biosciences) flow cytometer and FlowJo analysis software (Tree Star, Inc.).

### Cytosolic Free Ca^2+^ Imaging

MDA-MB-231 and PC-3 cells were seeded onto 22 mm square glass coverslips and then incubated with 6 μM CMR for 6–24 h. After washing with PBS, the cells were incubated with 4 μM Fura-2/AM in Krebs-HEPES buffer (10 mM HEPES, 135 mM NaCl, 6 mM KCl, 2 mM CaCl_2_, 1.2 mM MgCl_2_, and 10 mM glucose at pH 7.4) for 1 h at room temperature in the dark. Live cells were imaged in the Ex340/380/Em505 spectrum using an Olympus FV1000 confocal microscope (Olympus Corp.).

### Mitochondrial Membrane Potential Assay

The effect of CMR treatment on mitochondrial membrane potential was assessed using a TMRE assay kit (Abcam, ab113852). Cells were seeded onto 96-well plates, incubated, and then exposed to a range of concentrations of CMR for 24 h with 6 replicates per condition. The medium was removed and replaced with 200 μl of culture medium containing 250 nM TMRE, and then the cells were incubated in the CO_2_ incubator at 37 °C for 1 h. After washing with 0.2% BSA in PBS, the absorbance was determined in the Ex549/Em575 spectrum with a DTX 880 fluorescence plate reader (Beckman Coulter).

### Reactive Oxygen Species Assay

Cells were seeded onto 6-well plates and treated with a range of concentrations of CMR for 24 h, or with 100 μM H_2_O_2_ and 5 mM NAC for 1 h to serve as a positive and negative control. The medium was removed and replaced with 2 ml of PBS containing 10 μM 2′,7′-dichlorodihydrofluorescein diacetate (H2DCFDA) (Life technologies, C6827) and cells were incubated in the CO_2_ incubator at 37 °C for 30 min in the dark. After washing with PBS, the production of ROS was determined in the Ex_488_/Em_525_ spectrum using a FACSCalibur (BD Biosciences) flow cytometer and FlowJo analysis software (Tree Star, Inc.).

### Calpain Activity Assay

The effect of CMR on calpain 1 and 2 protease activity was evaluated using a Calpain-GloTM protease assay kit (Promega, G8501). Briefly, MDA-MB-231, PC-3 and LNCaP cells were seeded into 24-well plates and then treated with a range of concentrations of CMR for 24 h with 6 replicates per condition. After washing with PBS, the cells were lysed, Suc-LLVY-GloTM substrate was added to the cell lysates, and bioluminescence was measured with a microplate luminometer (Promega, E6501).

### Fluorescent quantitative PCR (Q-PCR)

Cellular RNAs were extracted using ExCellenCT Lysis Kit (ABM) according to the manufacturer’s instructions. Briefly, Cells were seeded into 96-well plates at 3 × 10^4^ cells/well. After CMR treatment for 48 h, remove the medium and wash the cells with 50 μl cold PBS. 1 μl Protease and 50 μl Lysis Solution were added to each well and treated for 10 minutes in incubator after mixing, followed by 1 μl inhibitor of Protease and 5 μl Stop Solution to end the reaction by incubating at room temperature and measured the RNA concentration. Reverse transcription uses 5 × All-In-One RT MasterMix (ABM) according to the manufacturer’s instructions in the non-RNase environment. The reverse transcription system contained 2 μl 5 × All-In-One RT MasterMix, 13 μl Nuclease-free water, 5 μl total RNA (2 RNA (2 water, 5 μx, 13 μ in the non-RNase enviro°C, 15 minutes, −42 °C, 30 minutes, −85 °C, 5 minutes, −4 °C. After the reverse transcription, fluorescent quantitative PCR was applied with 0.3 μL Primer-F (10 μM), 0.3 μL Primer-R (10 μM), 0.6 μl cDNA (10 ng/μl), 5 μl EvaGreen and 3.8 μL H2O. The reaction conditions were the same as those of reverse transcription.

### *In vivo* study

Female athymic nude mice (Foxn1nu; 5–7 weeks of age; Harlan Laboratories, Indianapolis, IN) were group-housed under a constant photoperiod of 12 hours’ light and 12 hours’ dark, and were provided ad libitum with sterilized food and water. All experimental procedures using live animals were conducted in accordance with protocols approved by The Ohio State University Institutional Animal Care and Use Committee. Xenograft tumors were established by subcutaneously injecting 1 × 10^6^ MDA-MB-231 cells in a total volume of 0.1 mL of 50% Matrigel (BD Biosciences, 354234) in PBS. To assess the effect of CMR on MDA-MB-231 tumor growth, mice with established tumors (mean starting tumor volume, 122.68 ± 24.91 mm^3^) were randomized into three groups (n = 6) that received 30 mg/kg CMR, 55 mg/kg CMR, or vehicle (10% DMSO, 20% polyethylene glycol and 5% Tween-80 in saline) based on the maximally tolerated dose (MTD) assay (Supplementary Fig. S16). All treatments were administered by intraperitoneal injection once daily for the entire study for a total of three weeks. Tumor volumes were calculated from caliper measurements using the standard formula of volume = width2 × length × 0.52^49^. Upon terminal sacrifice, the tumors were harvested, snap-frozen in liquid nitrogen, and stored at −80 °C until used to Western blot for biomarkers.

### Immunohistochemistry and H&E Staining

All tumor tissue samples were fixed in a buffered 10% formaldehyde solution, embedded in paraffin and sliced into 4 μm sections, which were either H&E stained or treated with specific antibodies for immunohistochemistry (IHC). For IHC, all specimens were first deparaffinized, immersed in sodium citrate for 20 min for antigen retrieval, and then incubated for 10 min in 3% H_2_O_2_ in methanol to block endogenous peroxidase activity. Next, the samples were blocked with 2% goat serum, incubated in anti-LC3-II antibody at 4 °C overnight, and then washed with PBS. The sections were coated with polymer enhancer and goat anti-mouse IgG, and incubated for 2 h at room temperature. After washing with PBS, the slices were developed for 30s using DAB. Finally, all of the samples were thoroughly washed and counterstained with hematoxylin.

### Immunofluorescence

After deparaffinization and antigen retrieval, the endogenous peroxidase activity of tumor tissues was quenched by immersing the slices in 3% H_2_O_2_ in methanol. After blocking with 5% BSA, the slices were incubated with primary antibodies against calpain1 and β-Actin. The slices were then washed with PBS three times and incubated with fluorophore-conjugated secondary antibodies. After another set of three washes in PBS, the slices were stained with DAPI (1 ug/ml). Finally, the slices were sealed and drop mounted to prevent quenching of fluorescence. Slides were then analyzed by confocal microscopy.

### Statistical Analysis

One-way ANOVA was used to test for statistical significance for all *in vitro* experiments using SPSS software (SPSS Inc., Chicago). The values with *p < *0*.*05* is considered statistically significant, **p < *0*.*01*.

## Electronic supplementary material

Supplementary Information
