# Supplementary material for: Chalcomoracin is a potent anticancer agent acting through triggering Oxidative stress via a mitophagy- and paraptosis-dependent mechanism
Source: Sci Rep. 2018 Jun 22;8:9566. doi: 10.1038/s41598-018-27724-3 (PMC6014977; doi:10.1038/s41598-018-27724-3)
Supplement: Supplementary file 1 — Supplementary Information [file 41598_2018_27724_MOESM1_ESM.pdf]

Chalcomoracin possesses as a potent anticancer agent by Triggering Oxidative Stress via a Mitophagy and Paraptosis dependent mechanism

Haote Han [#,1,2], Chih-Chien Chou [#,3], Ruyi Li [#,1], Jiangyun Liu[4], Lin Zhang[1,2], Wei Zhu,[1,2], Jin Hu[1,2], Bingxian Yang [1,2], Jingkui Tian[,1,2]*

[1]The Key Laboratory of Biomedical Engineering, Ministry of Education, Department of Biomedical Engineering, Zhejiang University, Hangzhou 310027, P.R. China

[2] Zhejiang-Malaysia Joint Research Center for Traditional Medicine, Zhejiang University, Hangzhou 310027, P.R. China

[3] Department of Radiation Oncology, Stanford University School of Medicine, Stanford, CA, United States of America

[4] A College of Pharmaceutical Sciences, Soochow University, Suzhou 215123, P.R. China

[#] Equally contributed to this study.

[*] Correspondence to:

Jingkui Tian, e-mail: tjk@zju.edu.cn; Tel.: +86-571-87951301; Fax: +86-571-87951676

Fig. S1

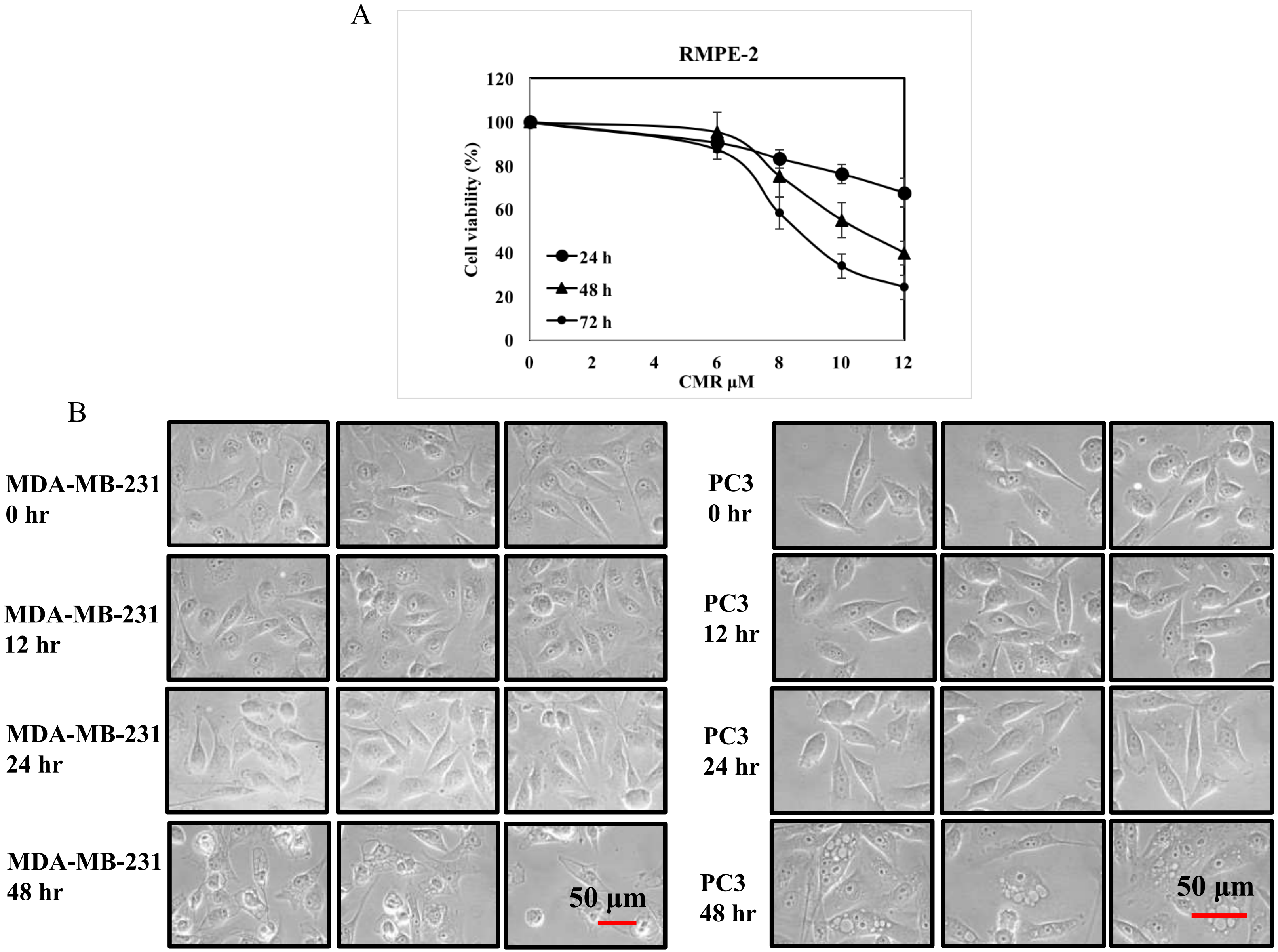

A
RMPE-2
Cell viability (%)
120
100
80
60
40
20
0
0
2
4
6
8
10
12
CMR μM
24 h
48 h
72 h
B
MDA-MB-231 0 hr
MDA-MB-231 12 hr
MDA-MB-231 24 hr
MDA-MB-231 48 hr
PC3 0 hr
PC3 12 hr
PC3 24 hr
PC3 48 hr
50 μm
50 μm

Fig. S2

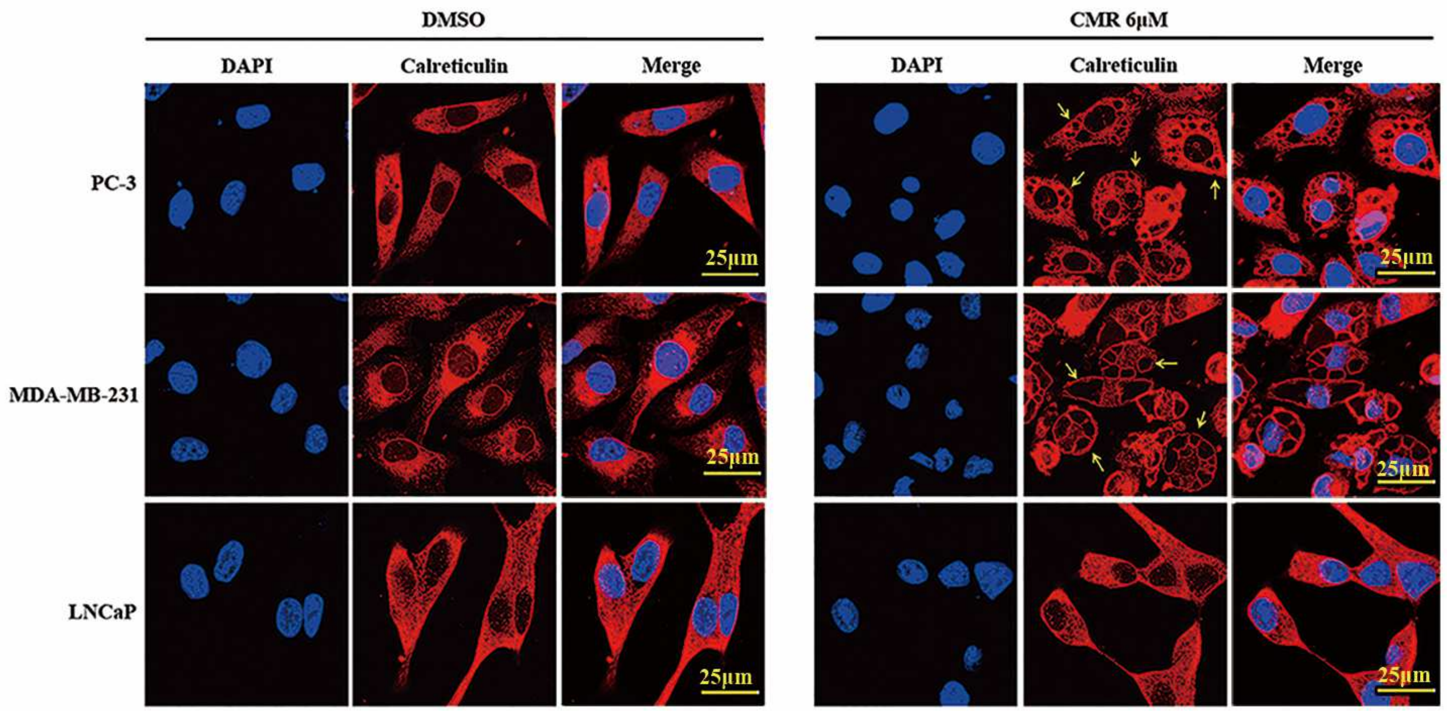
DMSO
CMR 6μM
DAPI
Calreticulin
Merge
DAPI
Calreticulin
Merge
PC-3
MDA-MB-231
LNCaP
25μm
25μm
25μm
25μm
25μm
25μm

Fig. S3

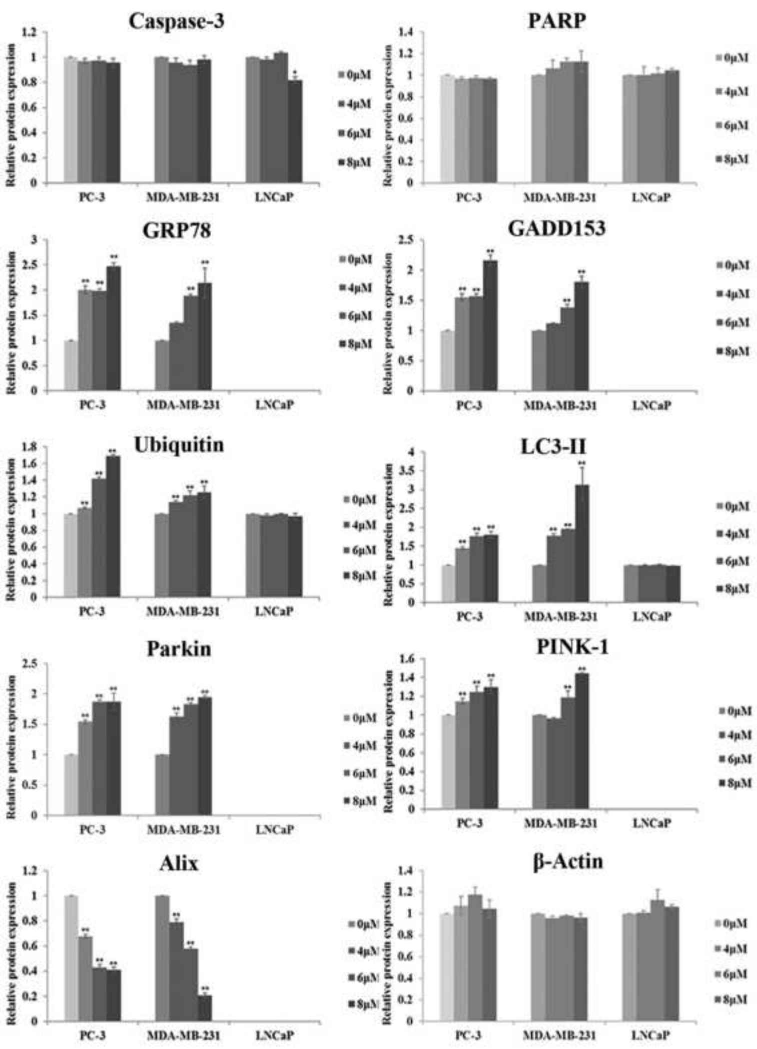

Caspase-3
PARP
GRP78
GADD153
Ubiquitin
LC3-II
Parkin
PINK-1
Alix
β-Actin
Relative protein expression
PC-3
MDA-MB-231
LNCaP
0μM
4μM
6μM
8μM

Fig. S4

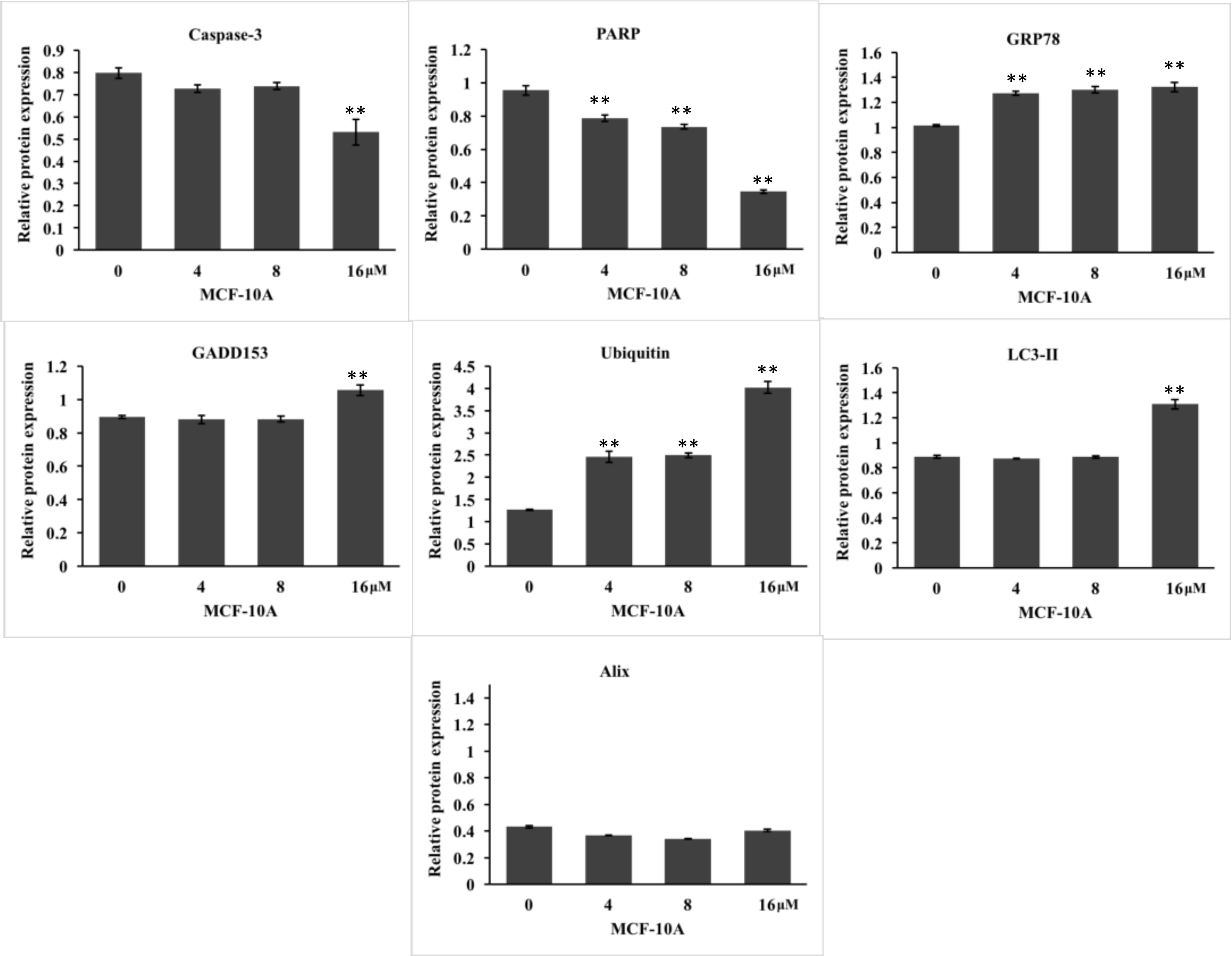
Caspase-3
PARP
GRP78
GADD153
Ubiquitin
LC3-II
Alix
Relative protein expression
0
4
8
16μM
MCF-10A
**

Fig. S5

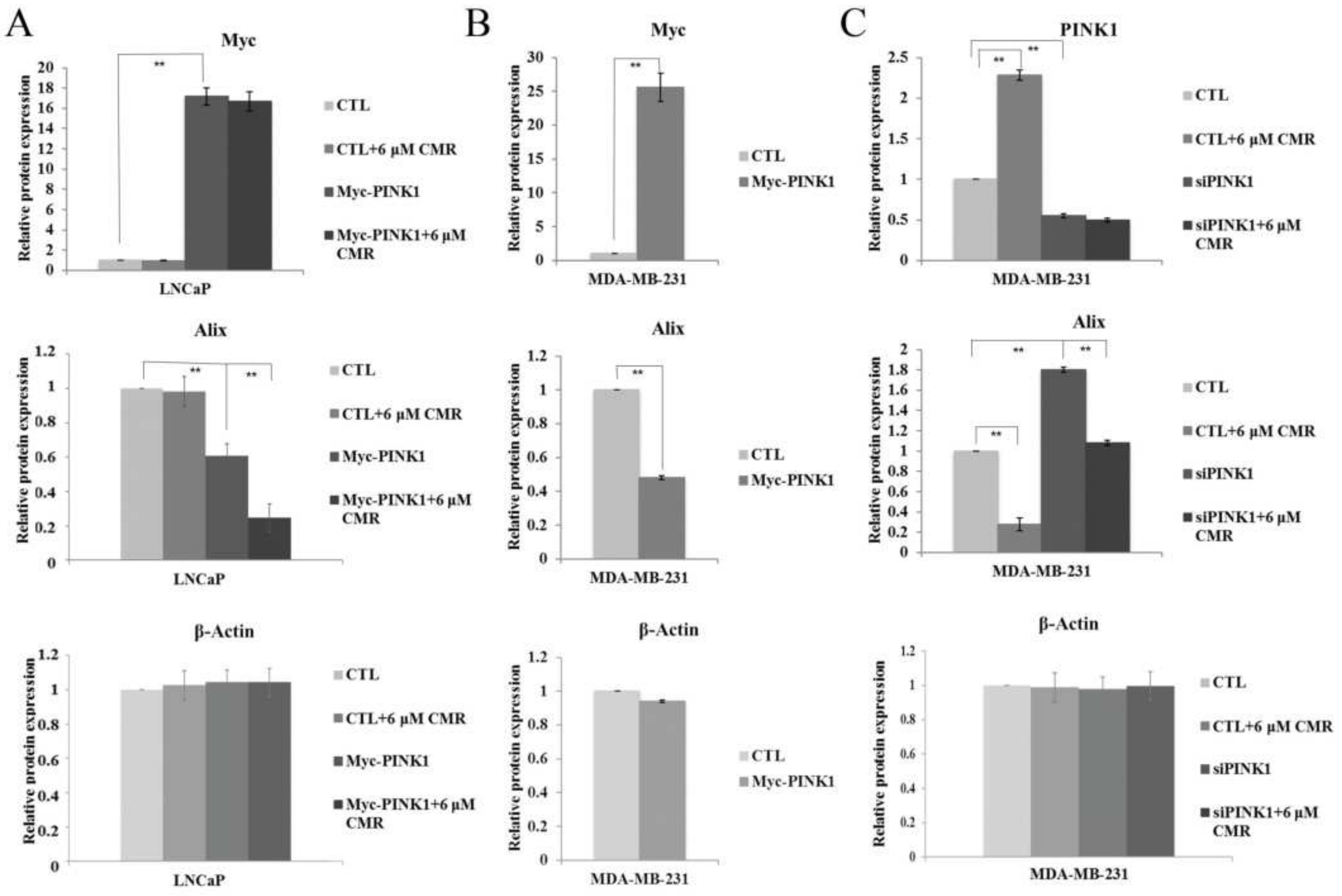
A
Myc
Relative protein expression
**
CTL
CTL+6 μM CMR
Myc-PINK1
Myc-PINK1+6 μM CMR
LNCaP
Alix
**
**
β-Actin
B
Myc
MDA-MB-231
CTL
Myc-PINK1
Alix
**
β-Actin
C
PINK1
**
**
CTL
CTL+6 μM CMR
siPINK1
siPINK1+6 μM CMR
MDA-MB-231
Alix
**
**
**
β-Actin

Fig. S6

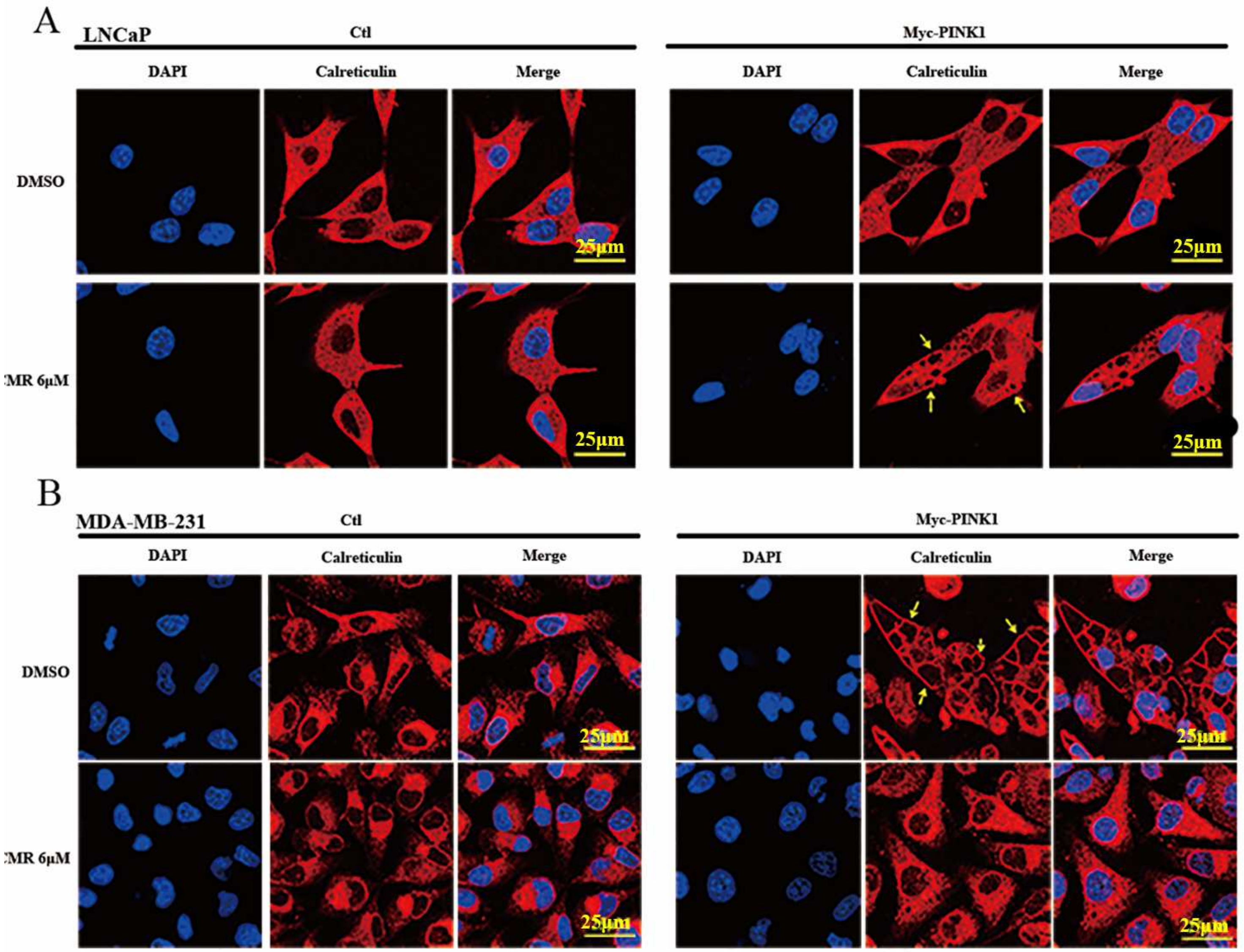
A
LNCaP
Ctl
Myc-PINK1
DAPI
Calreticulin
Merge
DMSO
MR 6μM
25μm
B
MDA-MB-231
Ctl
Myc-PINK1
DAPI
Calreticulin
Merge
DMSO
MR 6μM
25μm

Fig. S7

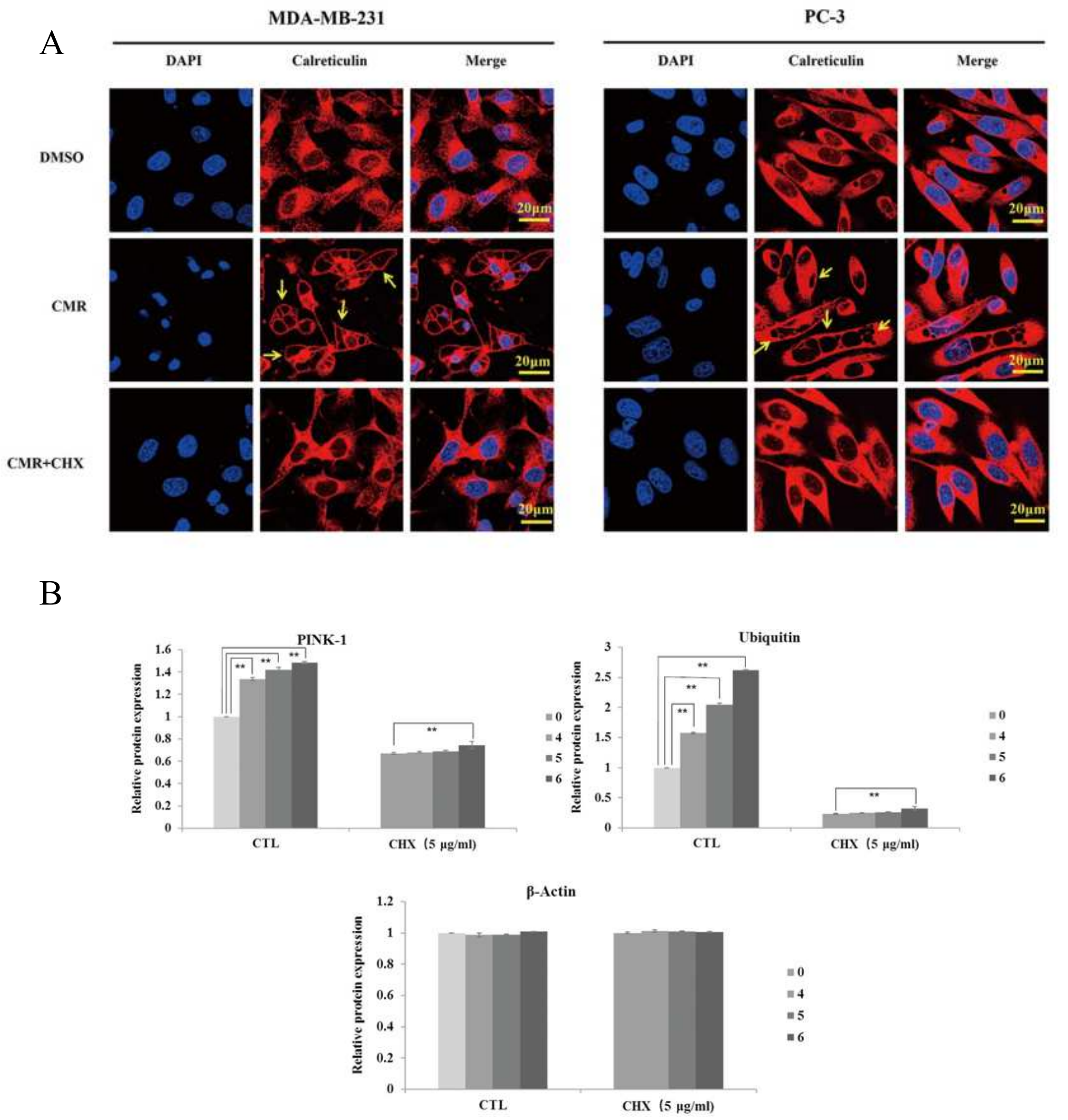

A
MDA-MB-231
PC-3
DAPI
Calreticulin
Merge
DMSO
CMR
CMR+CHX
20μm
B
PINK-1
Ubiquitin
β-Actin
Relative protein expression
CTL
CHX (5 μg/ml)
0
4
5
6
**

Fig. S8

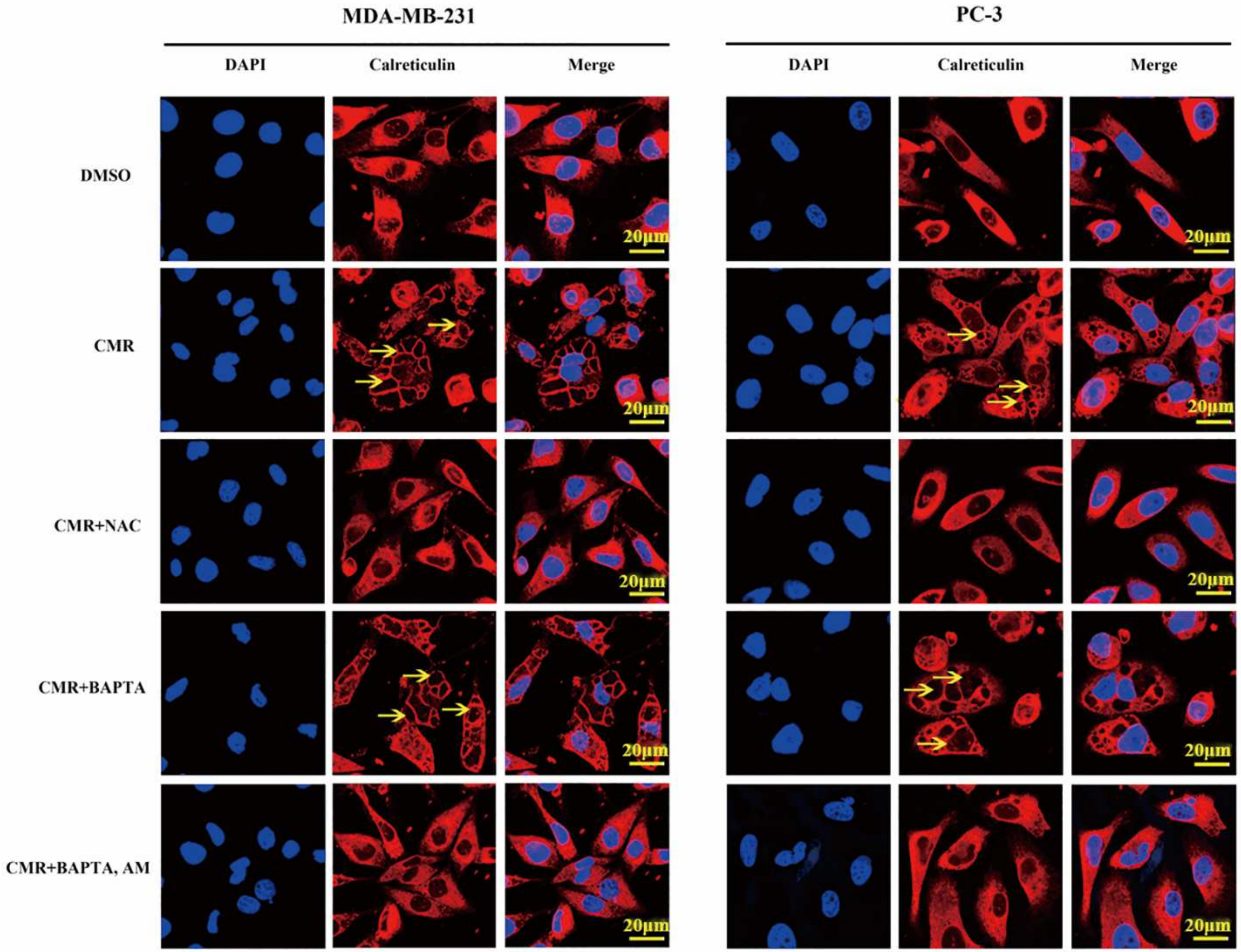
MDA-MB-231
PC-3
DAPI
Calreticulin
Merge
DMSO
CMR
CMR+NAC
CMR+BAPTA
CMR+BAPTA, AM
20μm

Fig. S9

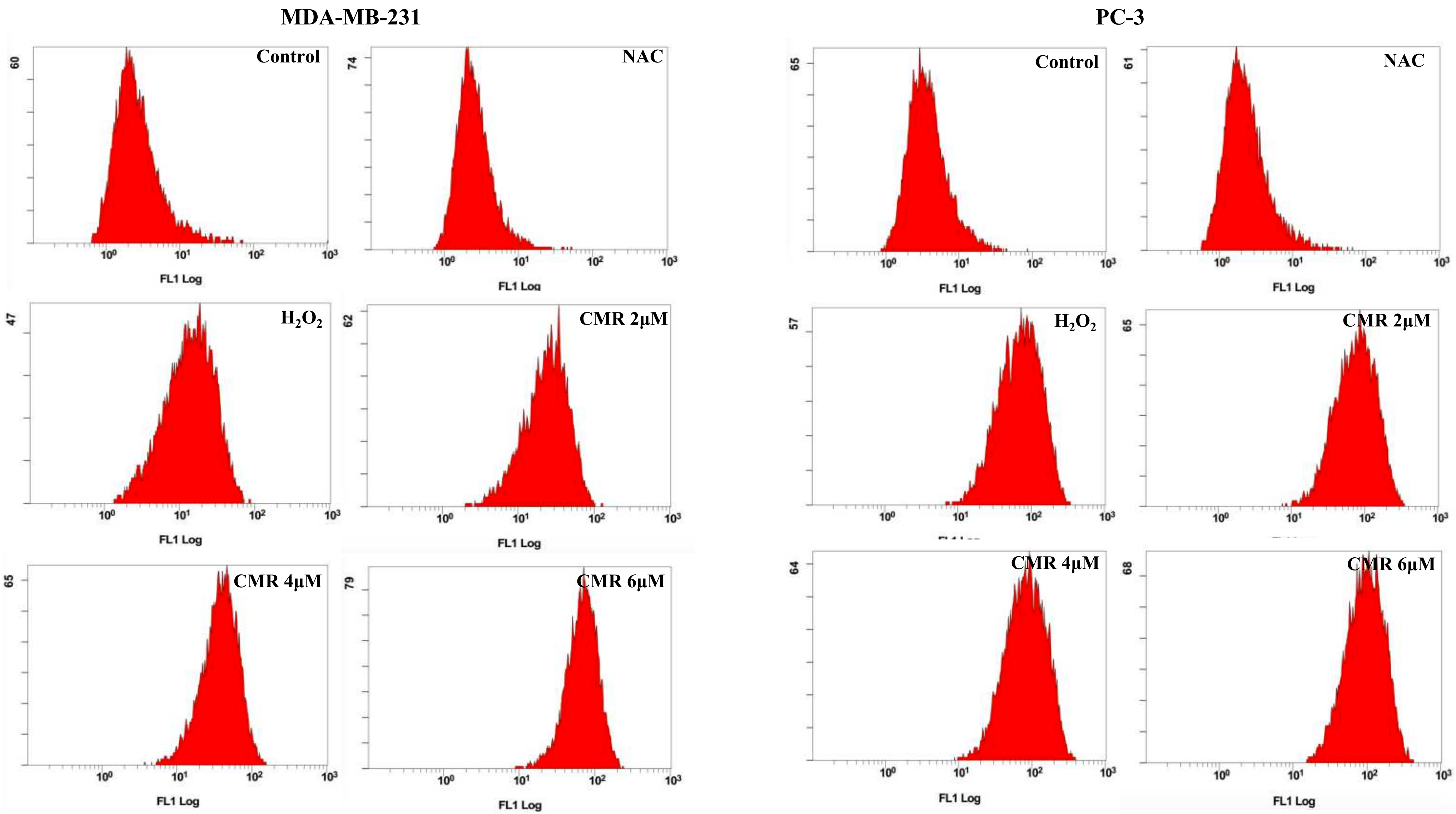

Fig. S10

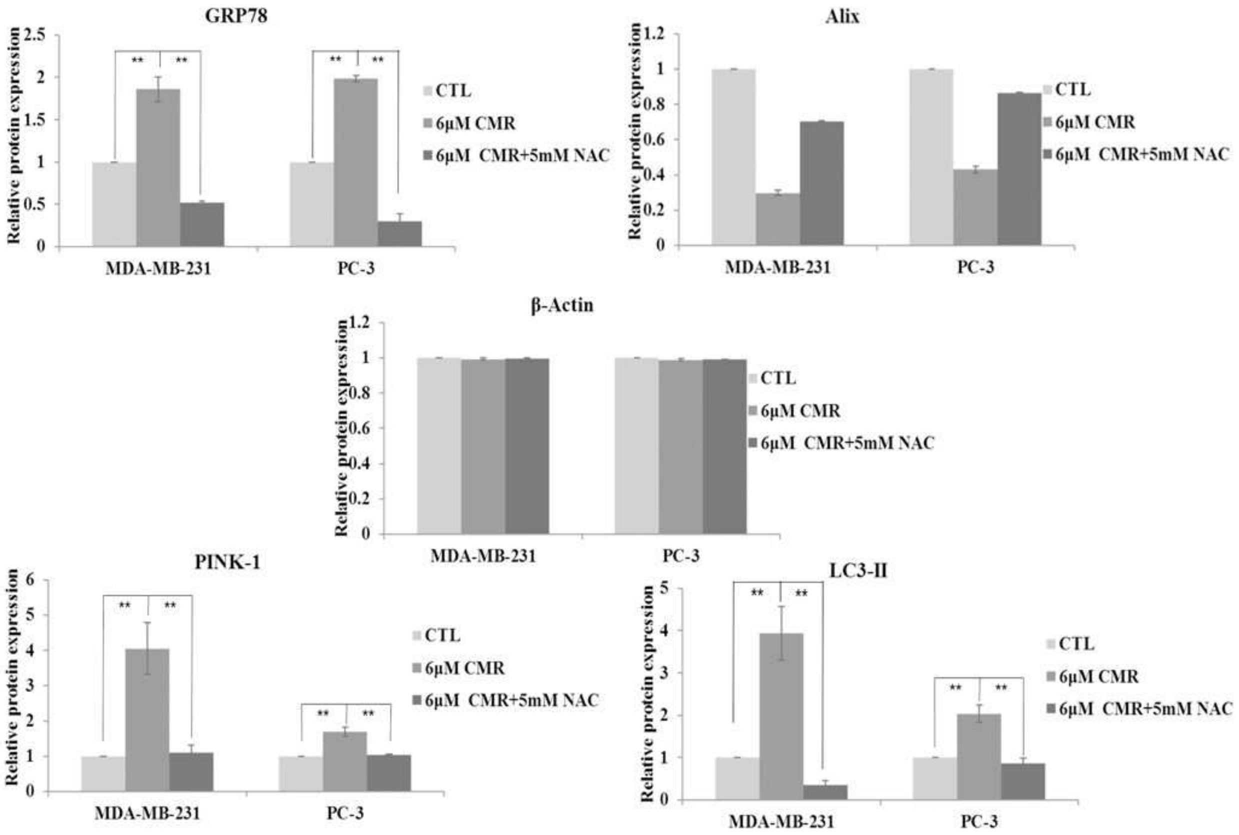
GRP78
Relative protein expression
2.5
2
1.5
1
0.5
0
**
**
MDA-MB-231
PC-3
CTL
6μM CMR
6μM CMR+5mM NAC
Alix
1.2
1
0.8
0.6
0.4
0.2
0
β-Actin
PINK-1
6
5
4
3
LC3-II

Fig. S11

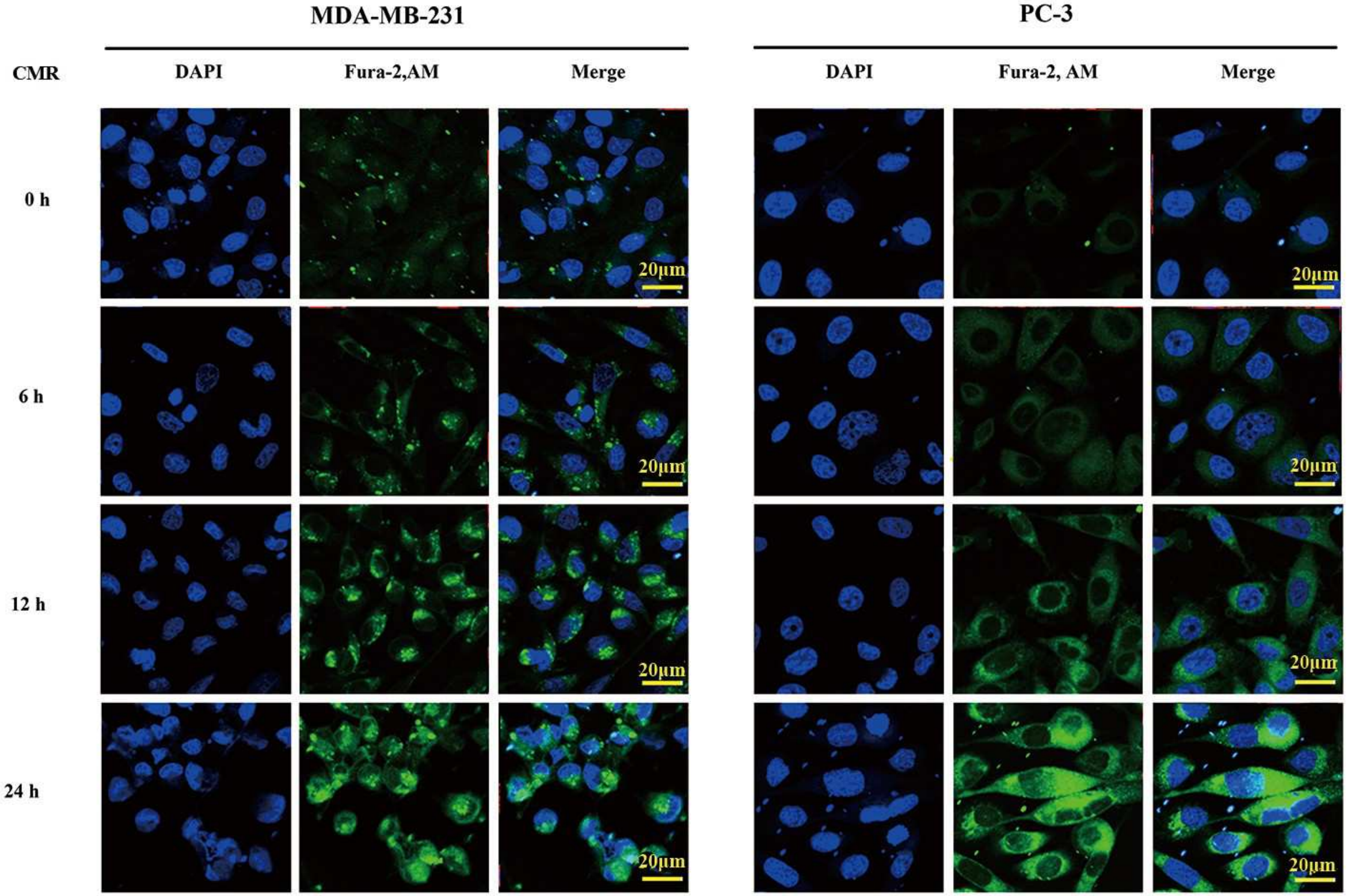
MDA-MB-231
PC-3
CMR
DAPI
Fura-2,AM
Merge
DAPI
Fura-2, AM
Merge
0 h
6 h
12 h
24 h
20μm

Fig. S12

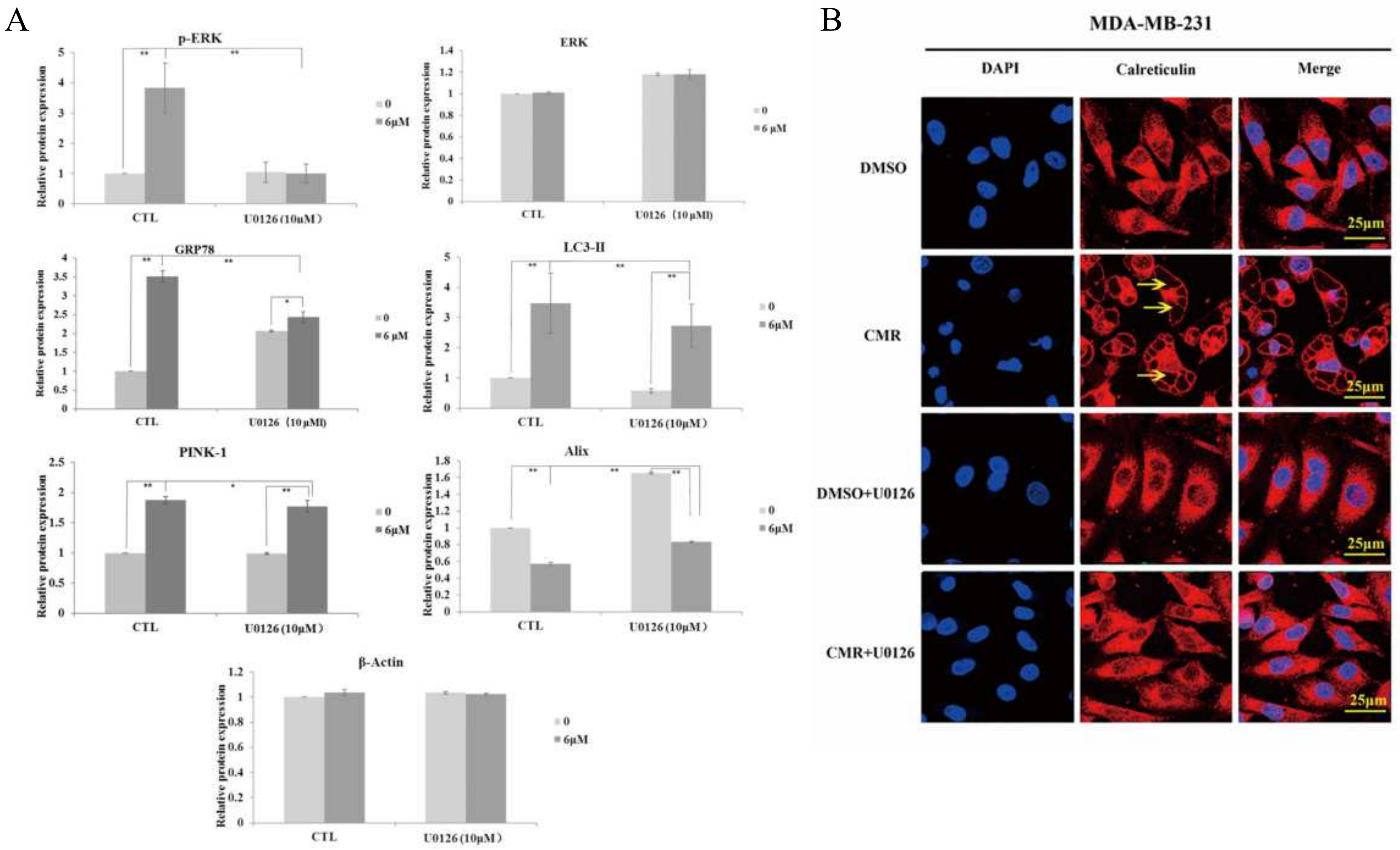
A
p-ERK
ERK
GRP78
LC3-II
PINK-1
Alix
β-Actin
Relative protein expression
CTL
U0126 (10μM)
0
6μM
B
MDA-MB-231
DAPI
Calreticulin
Merge
DMSO
CMR
DMSO+U0126
CMR+U0126
25μm

Fig. S13

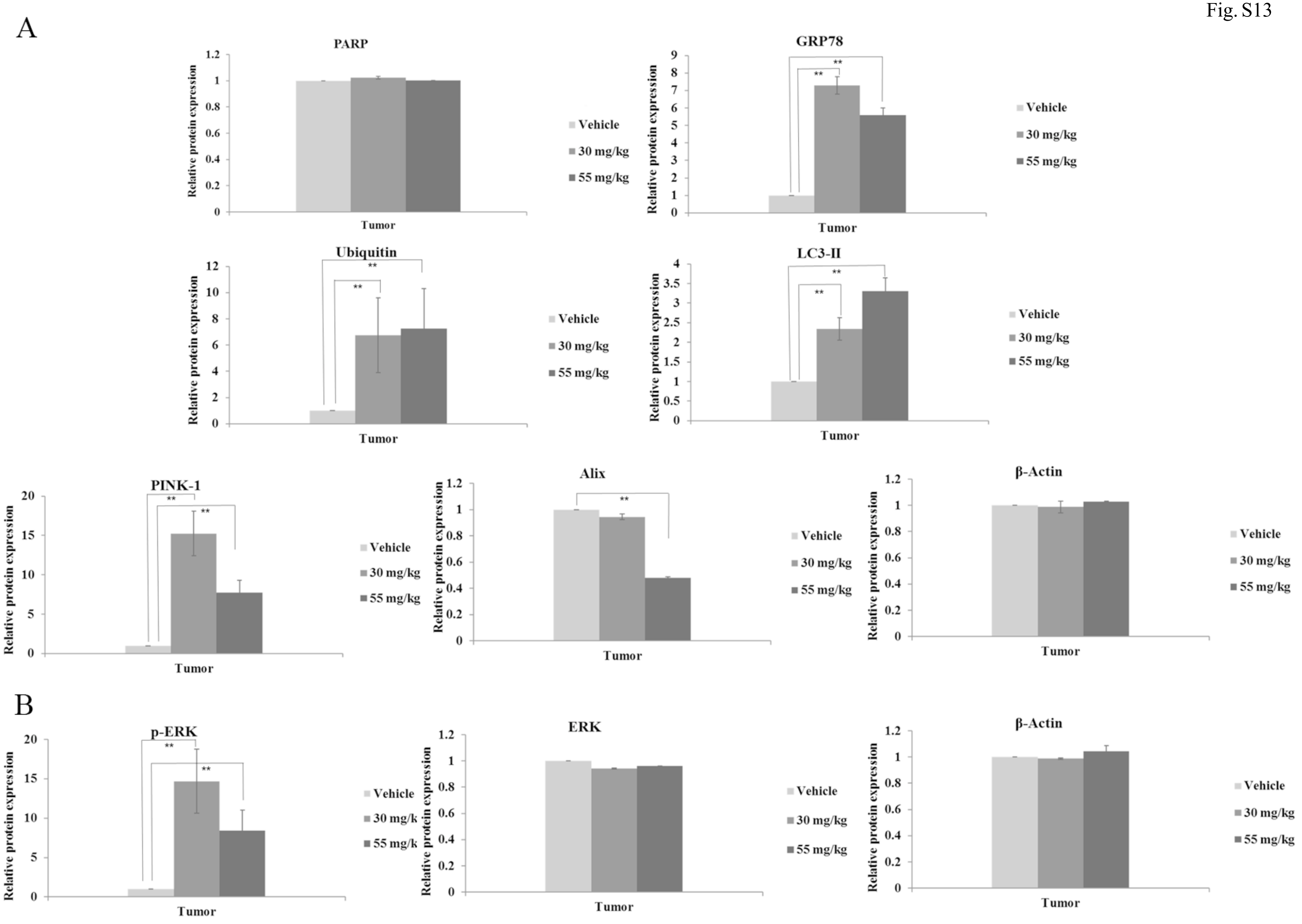
A
PARP
GRP78
Ubiquitin
LC3-II
PINK-1
Alix
β-Actin
B
p-ERK
ERK
β-Actin
Relative protein expression
Tumor
Vehicle
30 mg/kg
55 mg/kg
**

Fig. S14

A

B

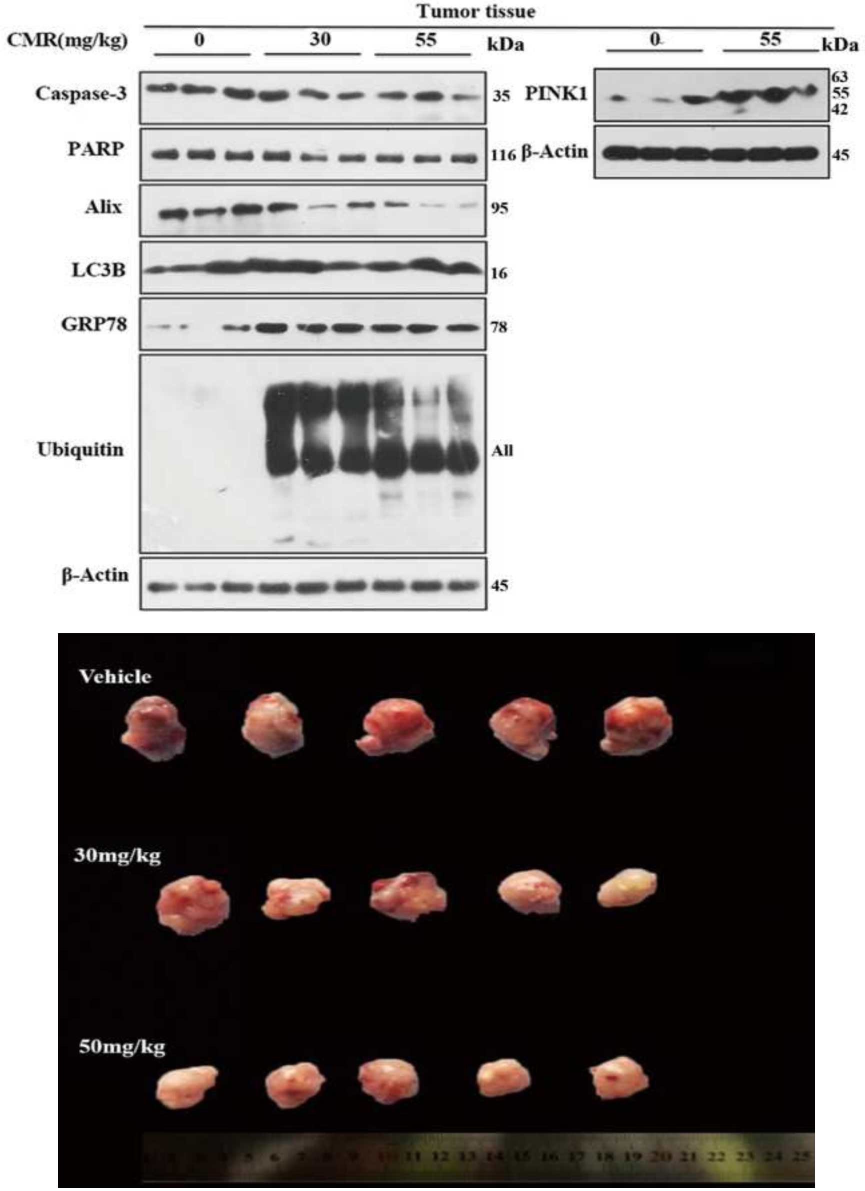
Tumor tissue
CMR(mg/kg)
0
30
55
kDa
Caspase-3
35
PARP
116
Alix
95
LC3B
16
GRP78
78
Ubiquitin
All
β-Actin
45
0
55
kDa
PINK1
63
55
42
β-Actin
45
Vehicle
30mg/kg
50mg/kg

Fig. S15

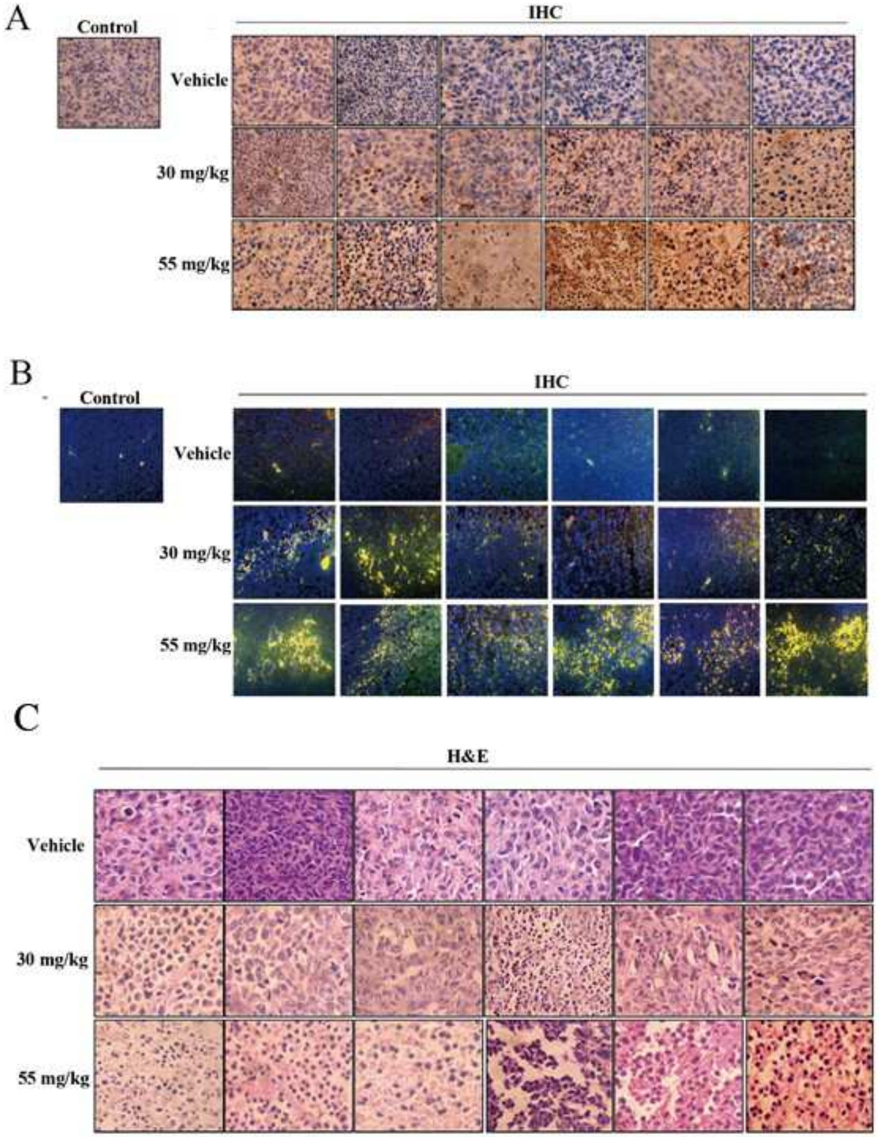
A
Control
IHC
Vehicle
30 mg/kg
55 mg/kg
B
Control
IHC
Vehicle
30 mg/kg
55 mg/kg
C
H&E
Vehicle
30 mg/kg
55 mg/kg

Fig. S16

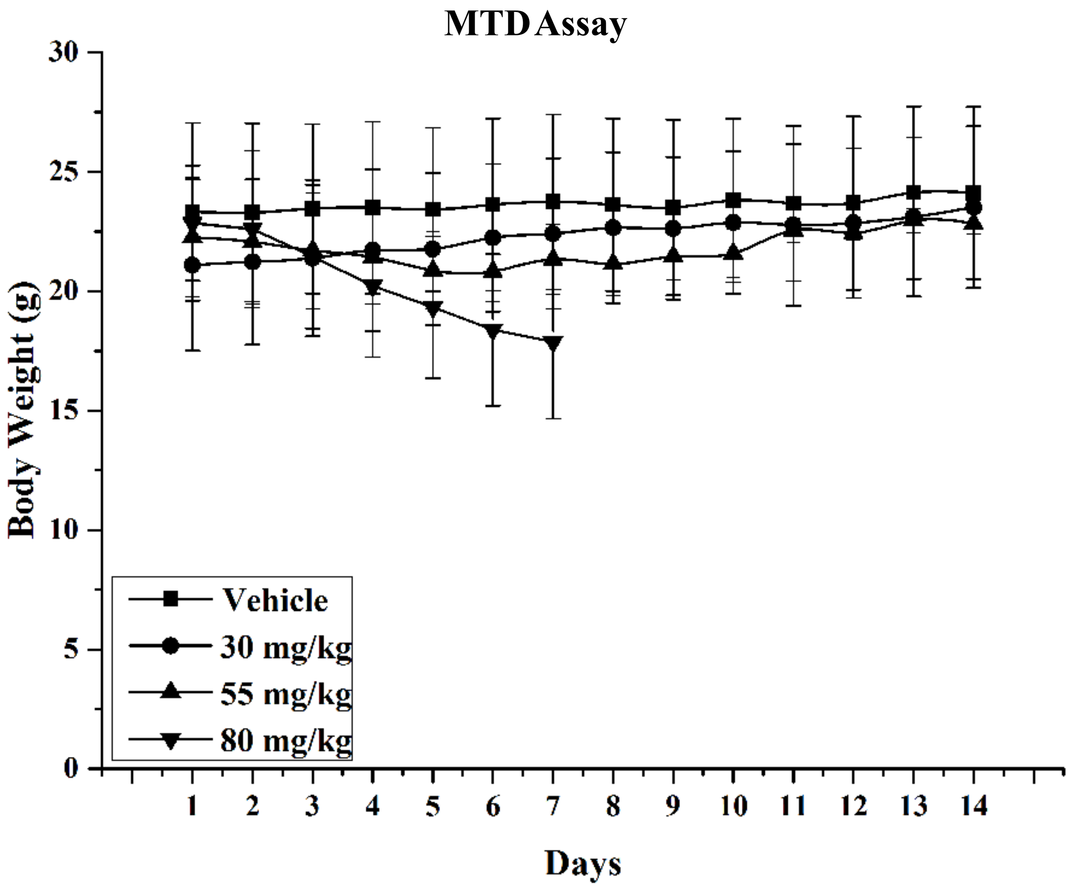

MTD Assay
Body Weight (g)
Days
Vehicle
30 mg/kg
55 mg/kg
80 mg/kg
0
5
10
15
20
25
30
1
2
3
4
5
6
7
8
9
10
11
12
13
14

Fig. S17

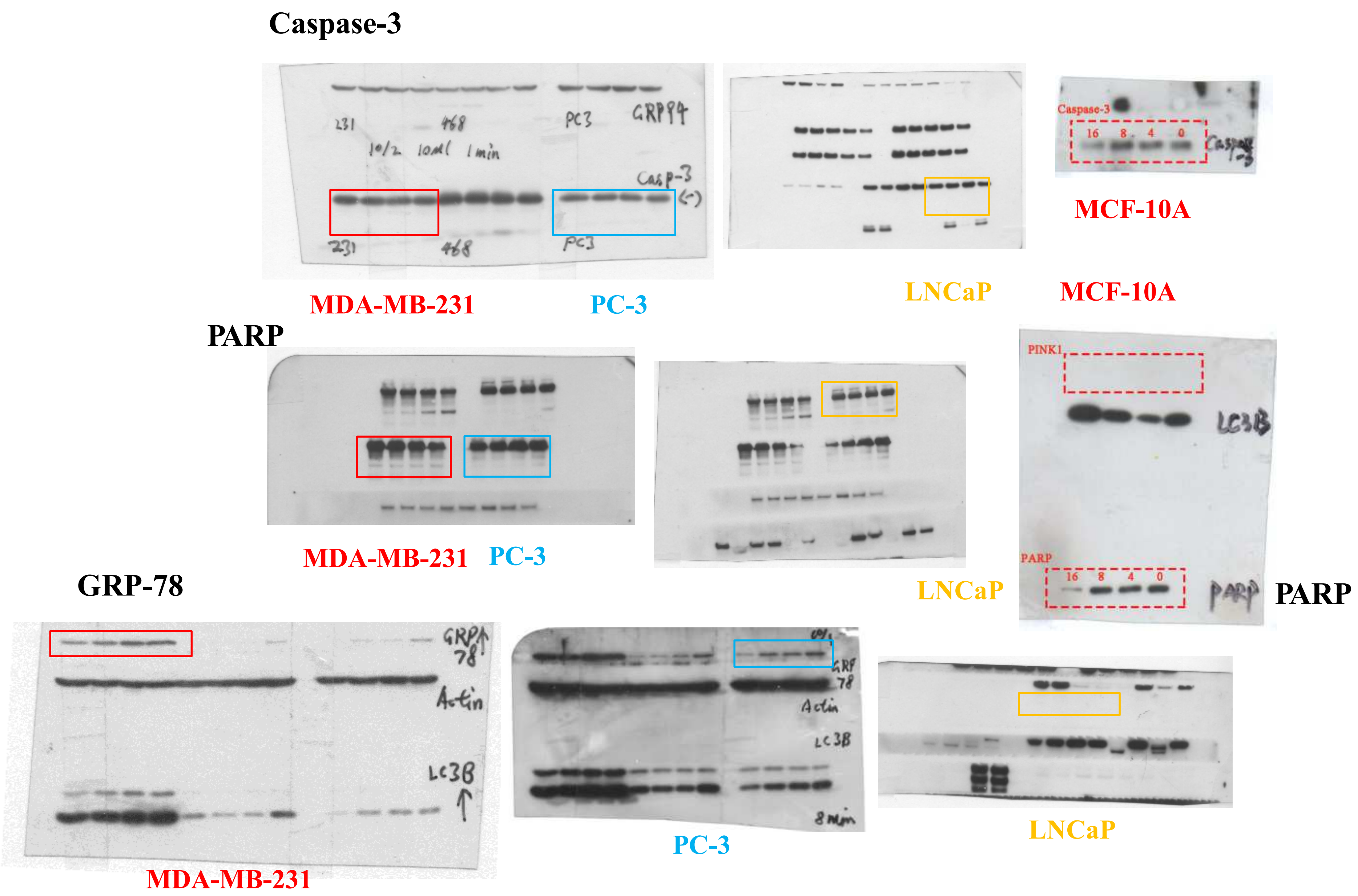
Caspase-3
231
468
PC3
GRP94
10/2
10ml
1min
Casp-3
(-)
MDA-MB-231
PC-3
LNCaP
Caspase-3
16
8
4
0
MCF-10A
MCF-10A
PARP
MDA-MB-231
PC-3
LNCaP
PINK1
LC3B
PARP
16
8
4
0
PARP
PARP
GRP-78
GRP
78
Actin
LC3B
MDA-MB-231
Actin
LC3B
8min
PC-3
LNCaP

Fig. S18

GADD-153

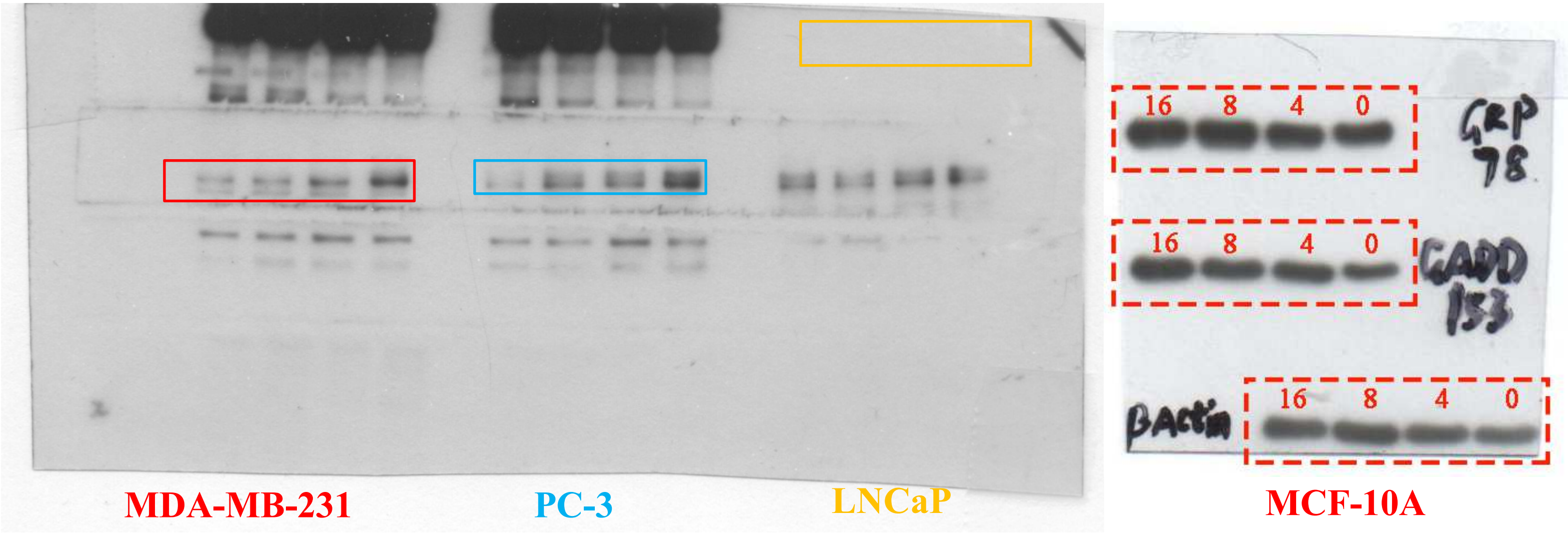

Ubiquitin

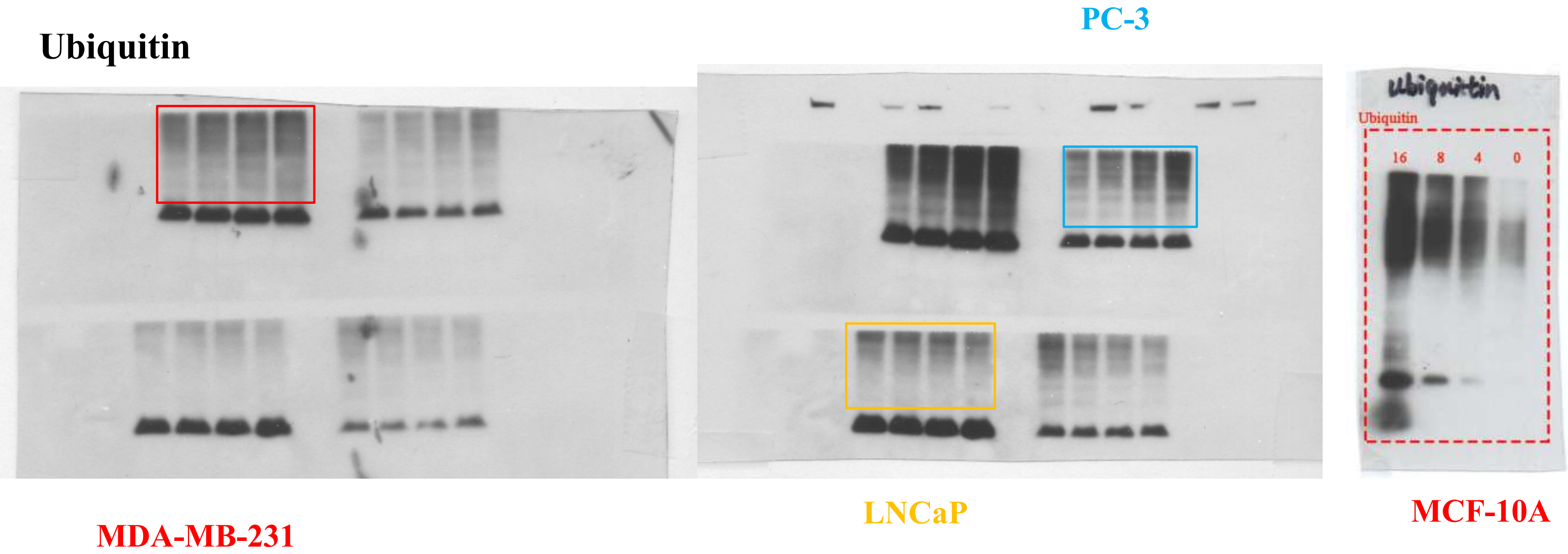

Fig. S19

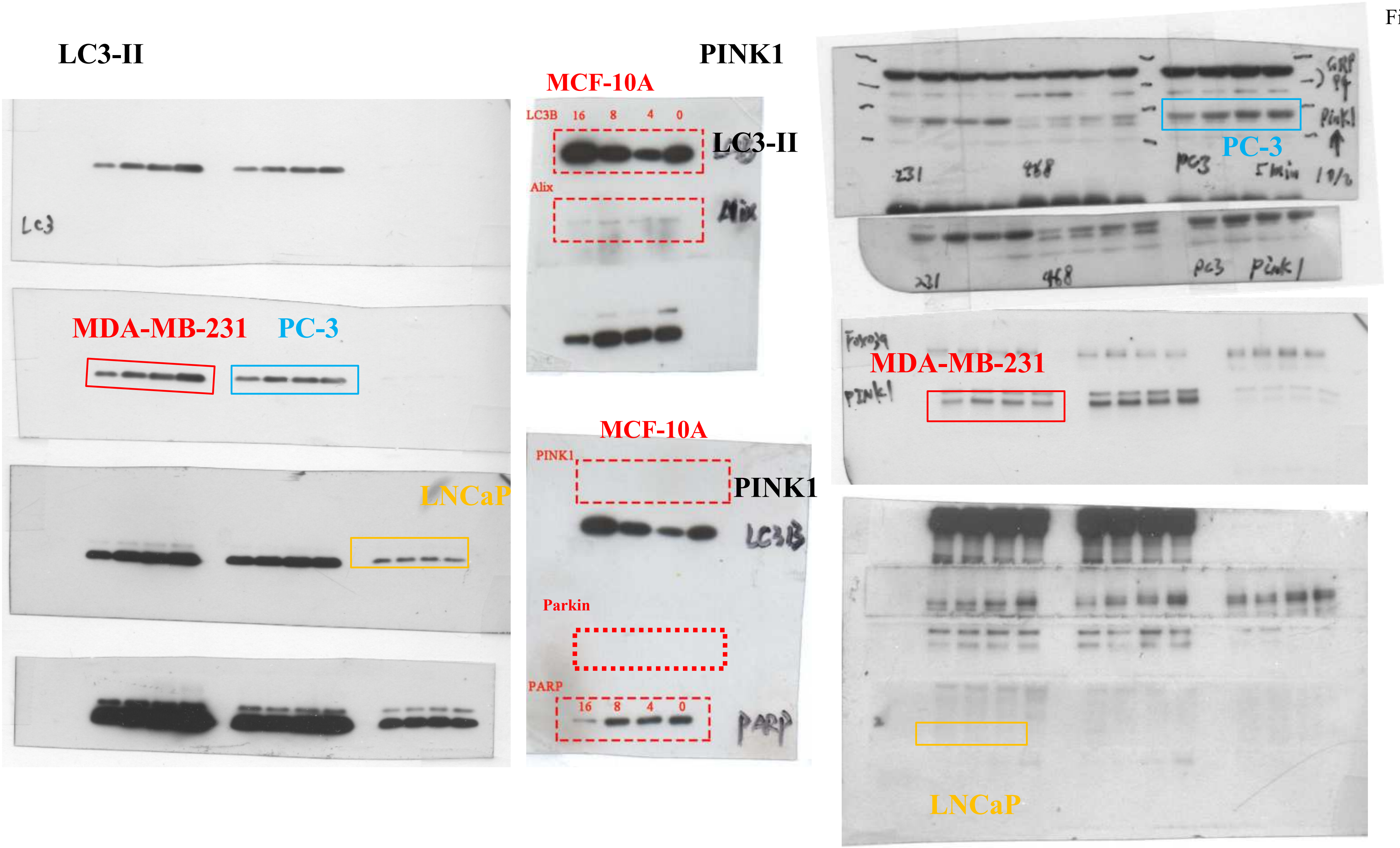
LC3-II
PINK1
MCF-10A
LC3B 16 8 4 0
LC3-II
Alix
MDA-MB-231
PC-3
LNCaP
MCF-10A
PINK1
PINK1
LC3B
Parkin
PARP
16 8 4 0
PARP
PC-3
MDA-MB-231
LNCaP

Fig. S20

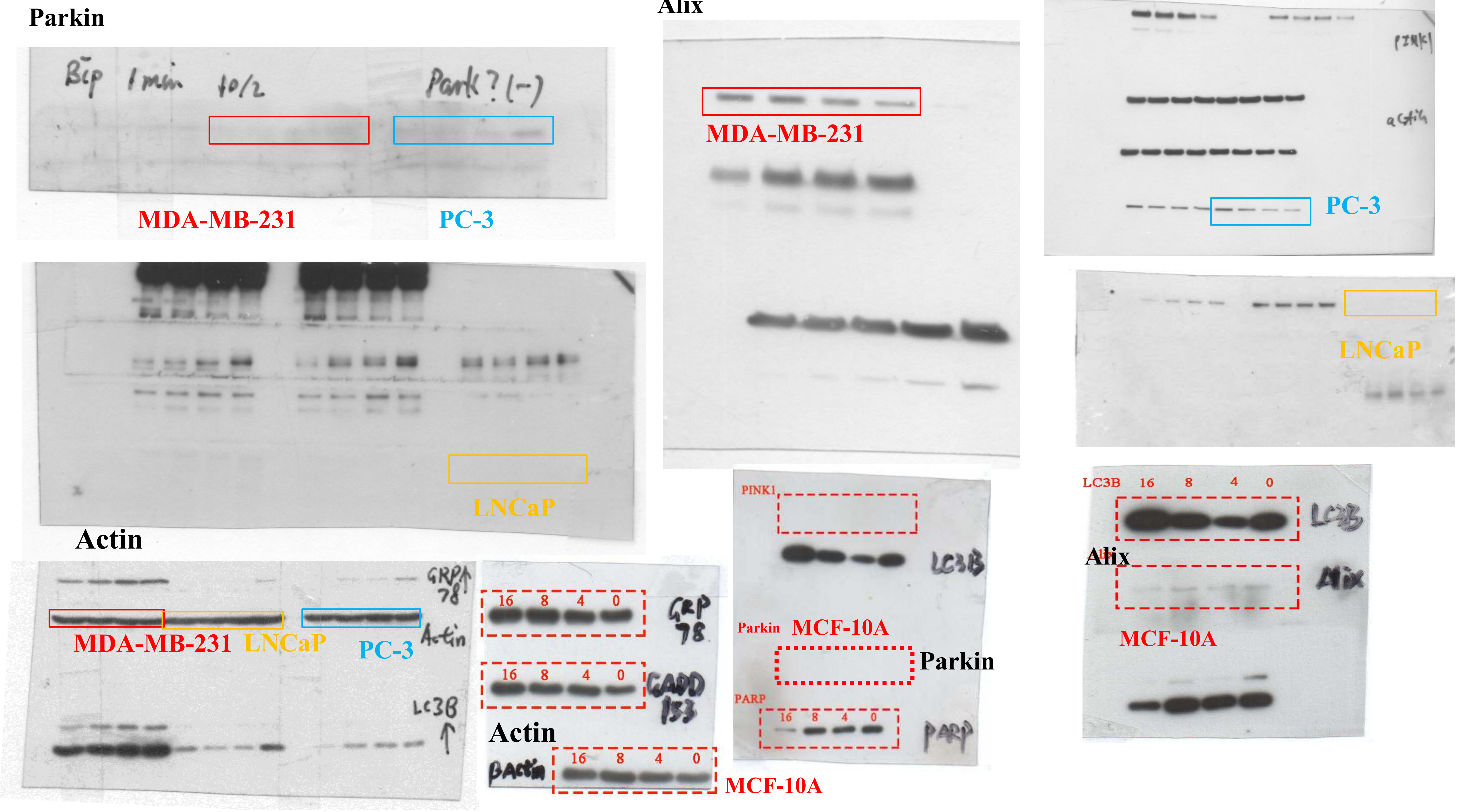
Parkin
MDA-MB-231
PC-3
LNCaP
Actin
MDA-MB-231
LNCaP
PC-3
Alix
MDA-MB-231
PC-3
LNCaP
16 8 4 0
16 8 4 0
Actin
16 8 4 0
MCF-10A
PINK1
Parkin
MCF-10A
Parkin
PARP
16 8 4 0
LC3B 16 8 4 0
Alix
MCF-10A

Fig. S21

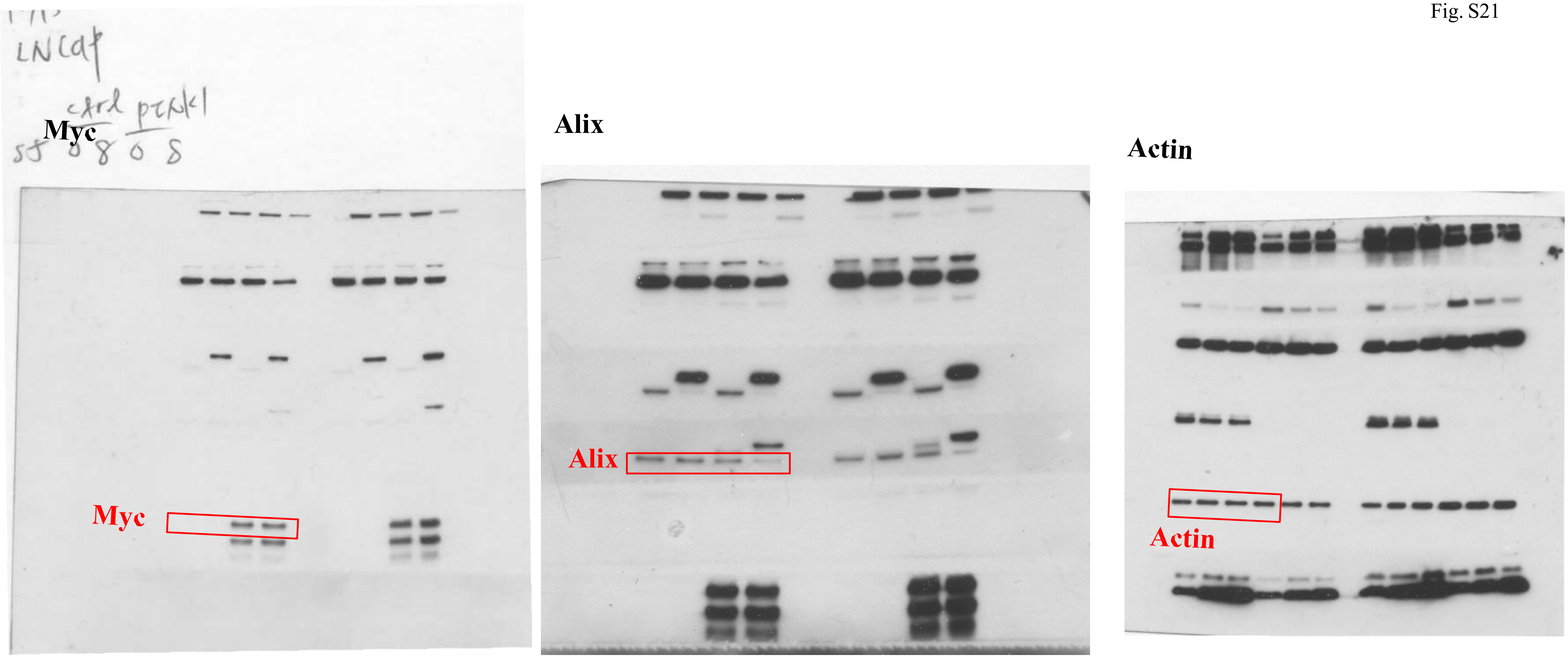

Fig. S22

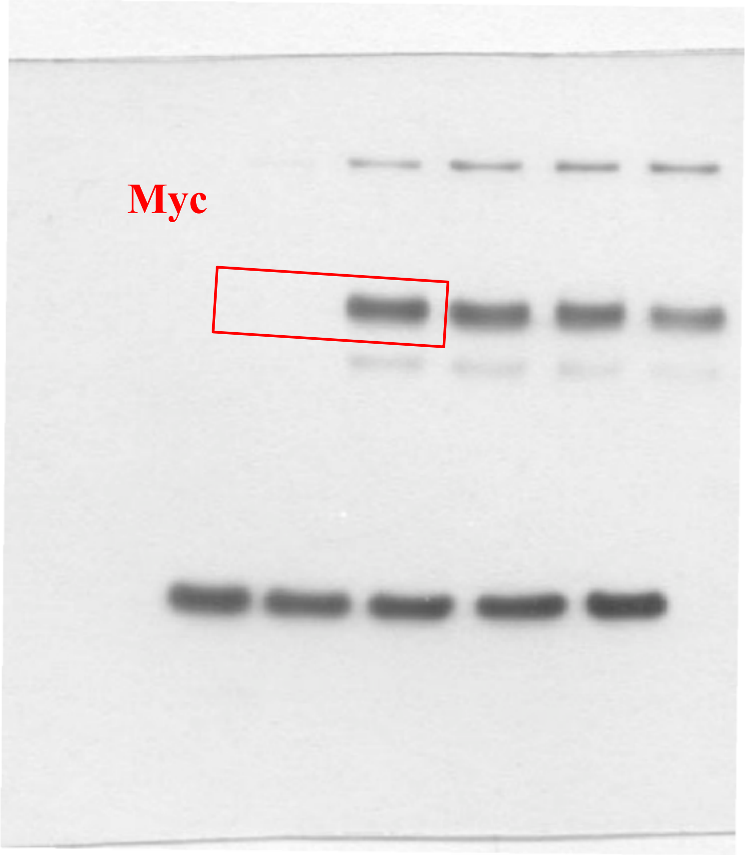

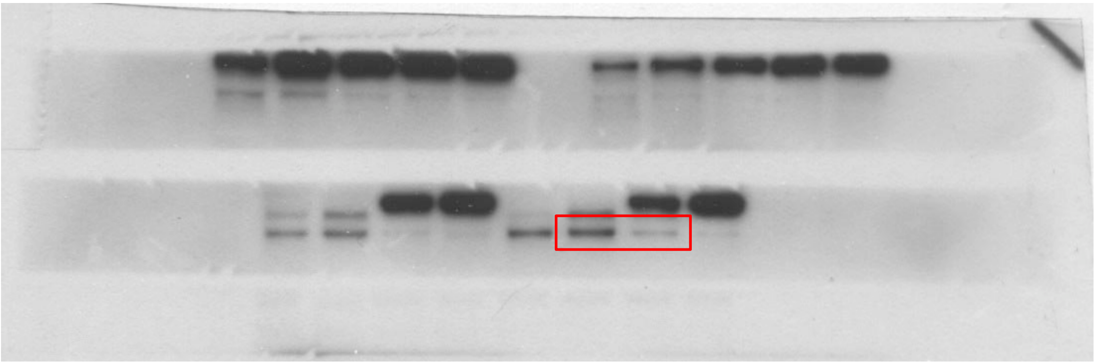

Alix

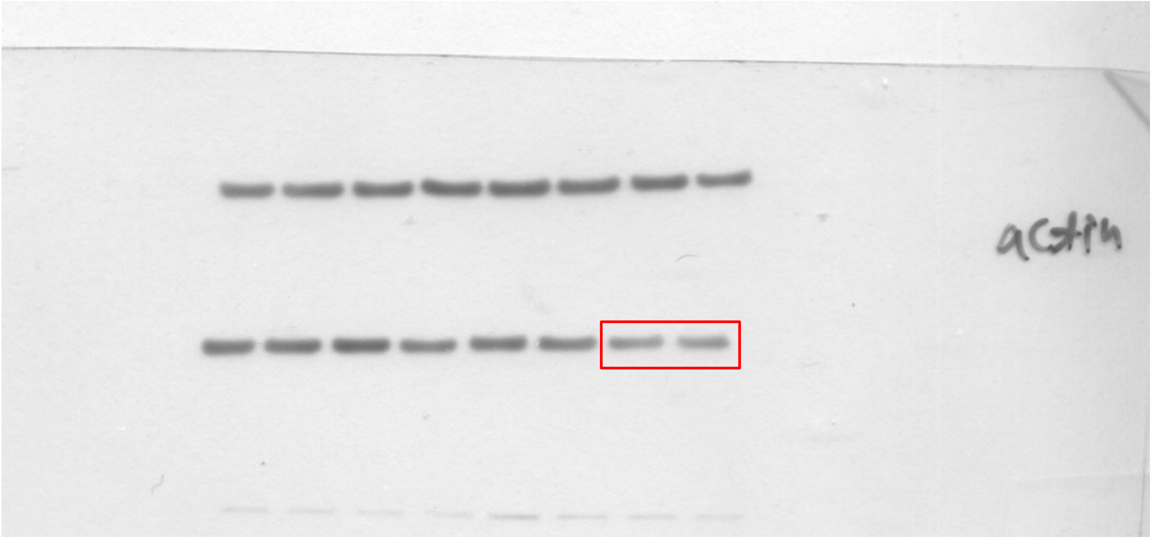

Actin

Fig. S23

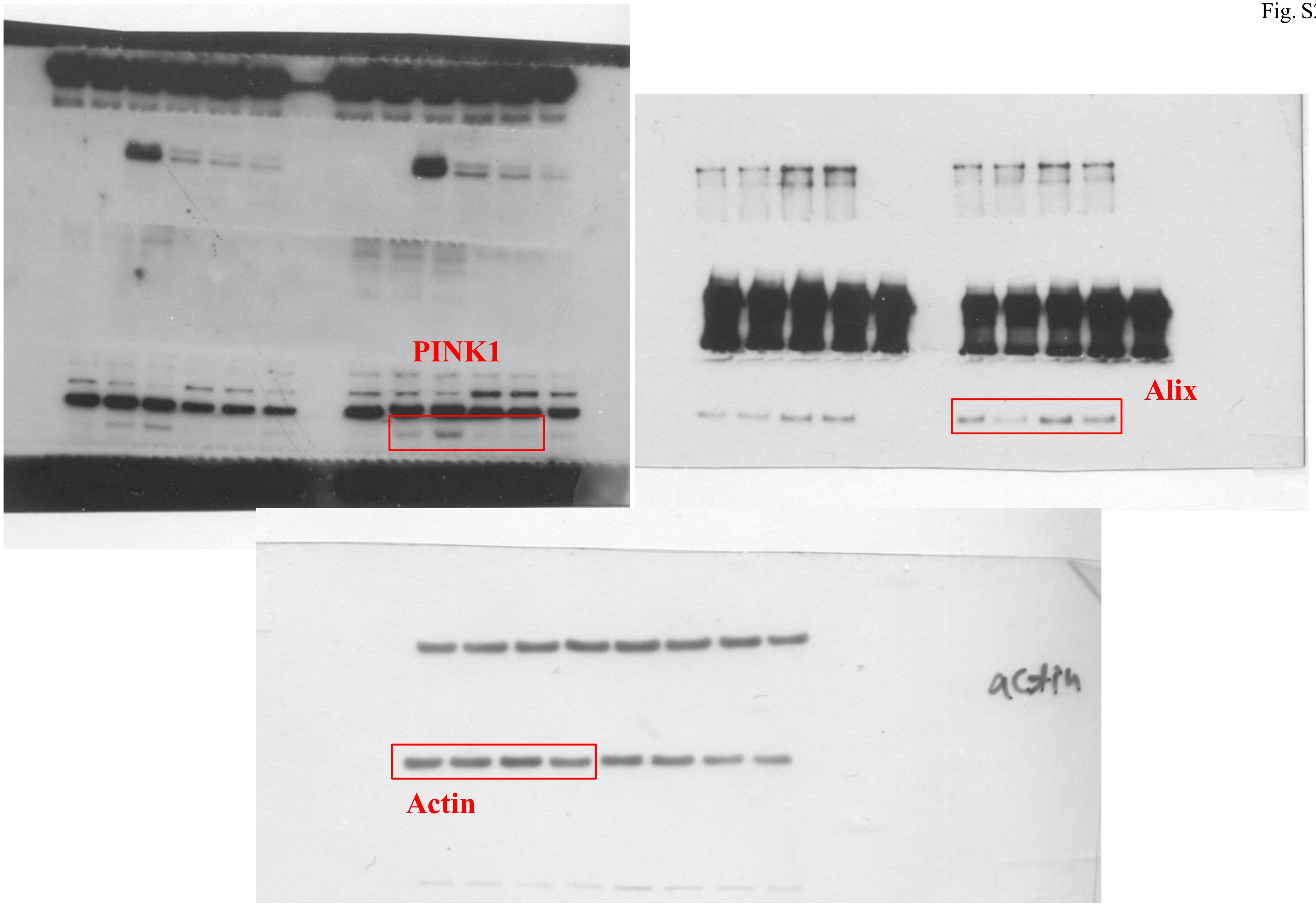

Fig. S24

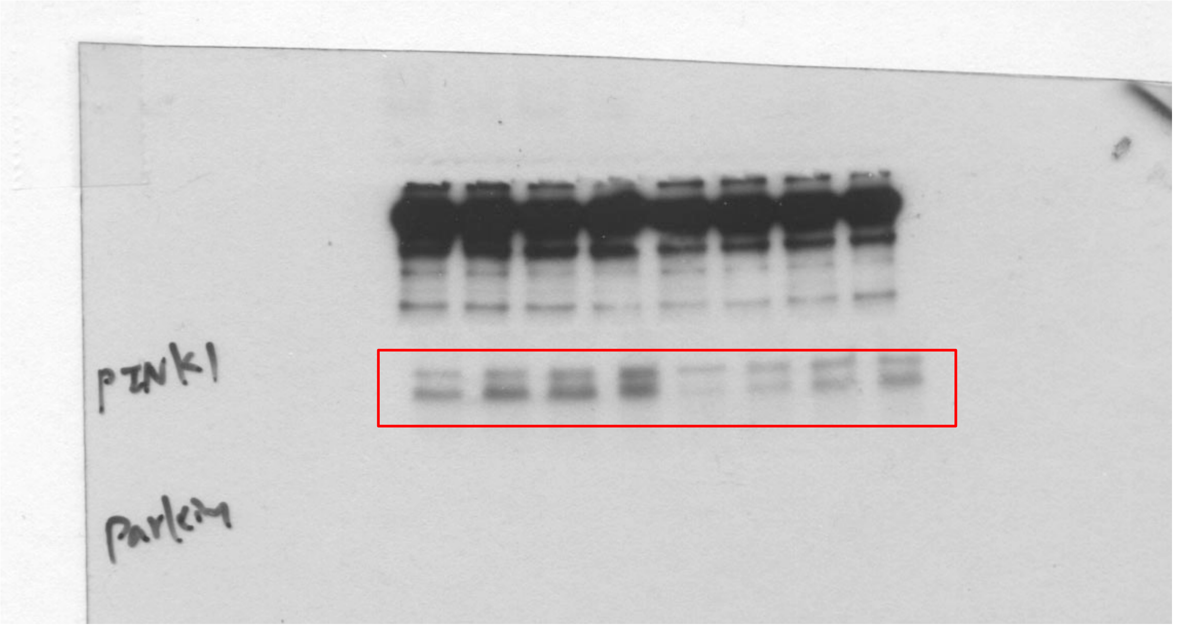

PINK1

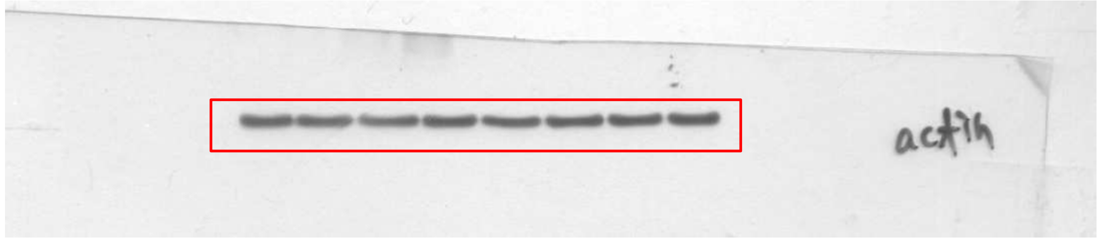

Actin

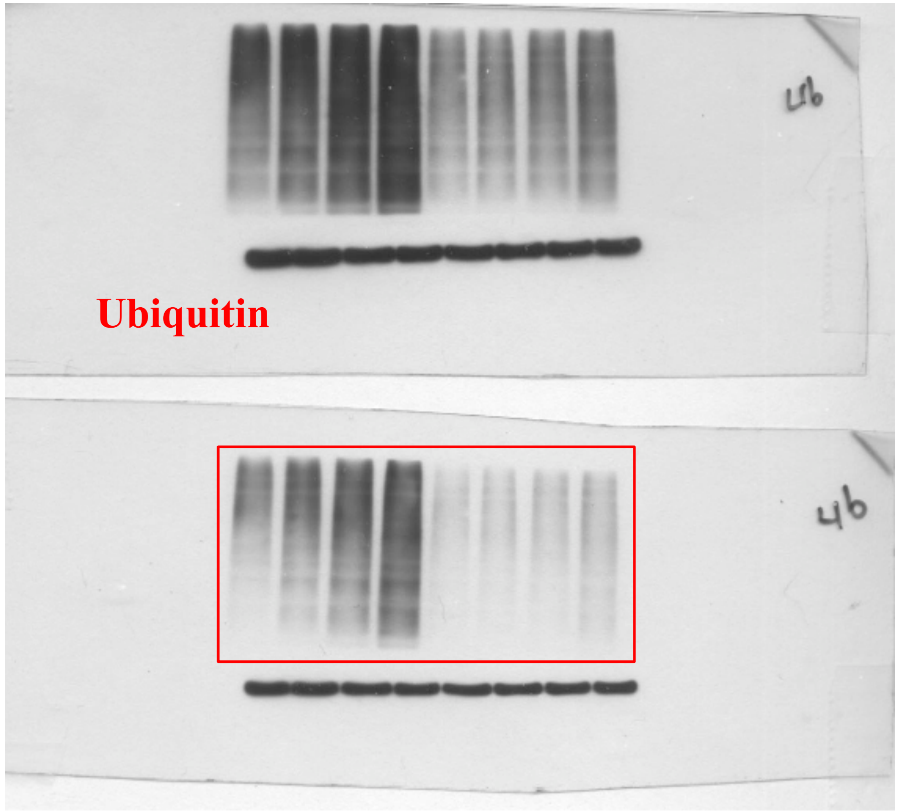

Ubiquitin

Fig. S25

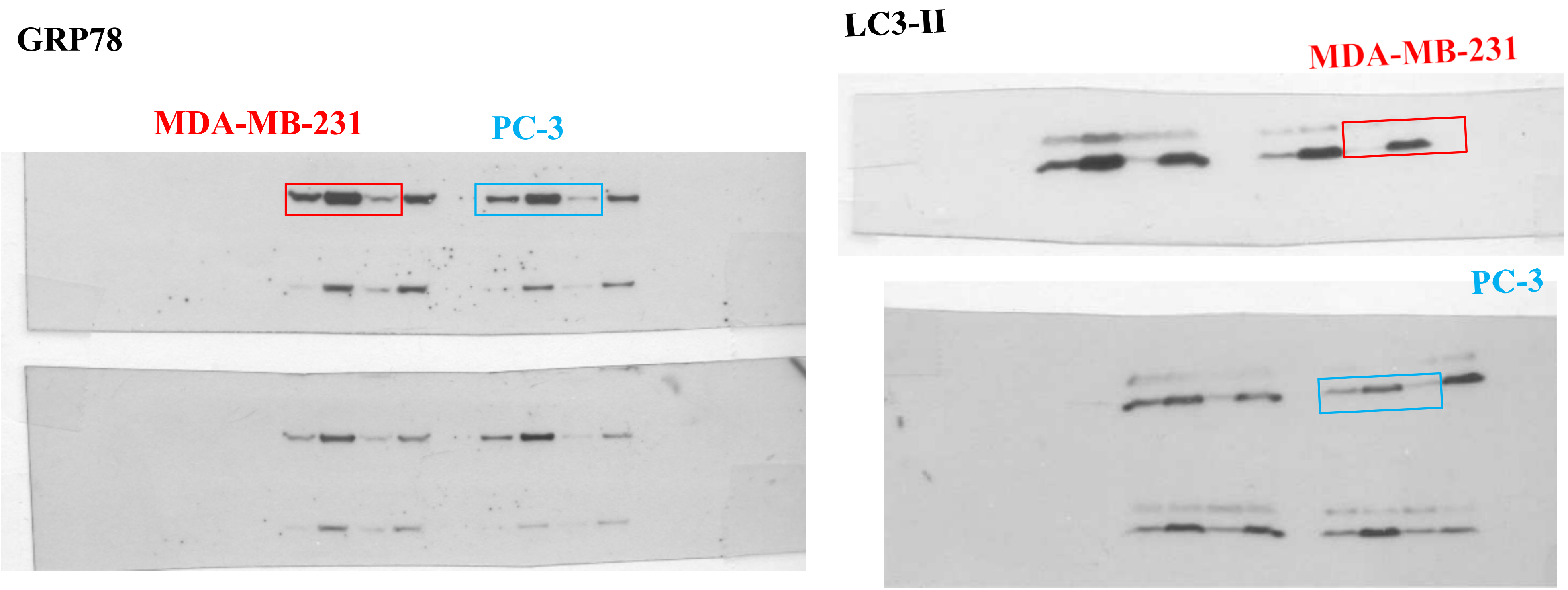

Fig. S26

PINK1

MDA-MB-231 PC-3

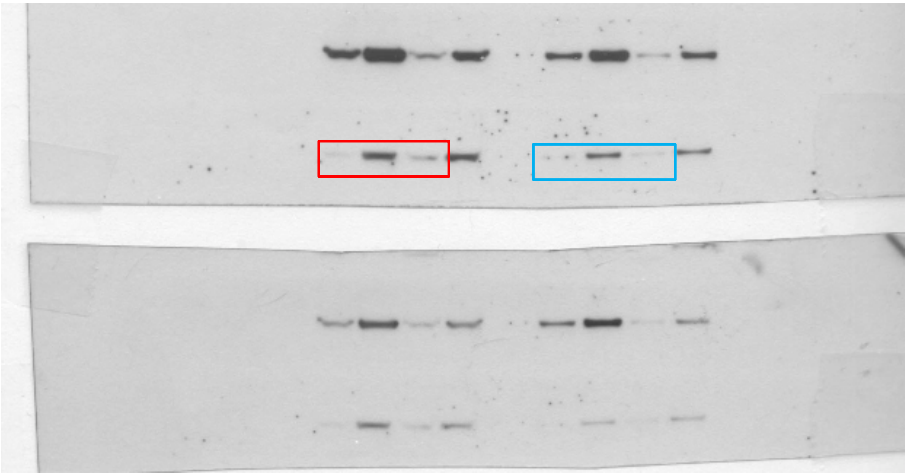

Alix

MDA-MB-231 PC-3

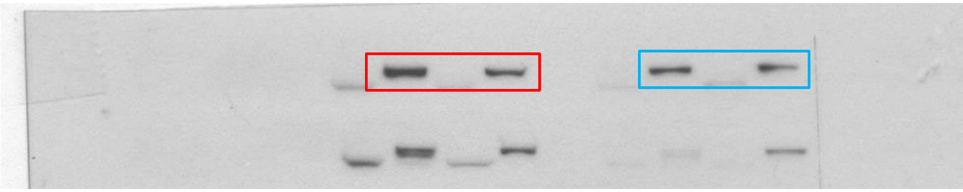

Actin

MDA-MB-231 PC-3

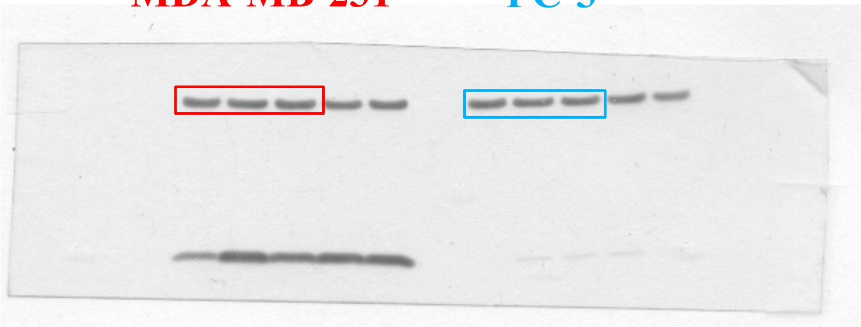

Fig. S27

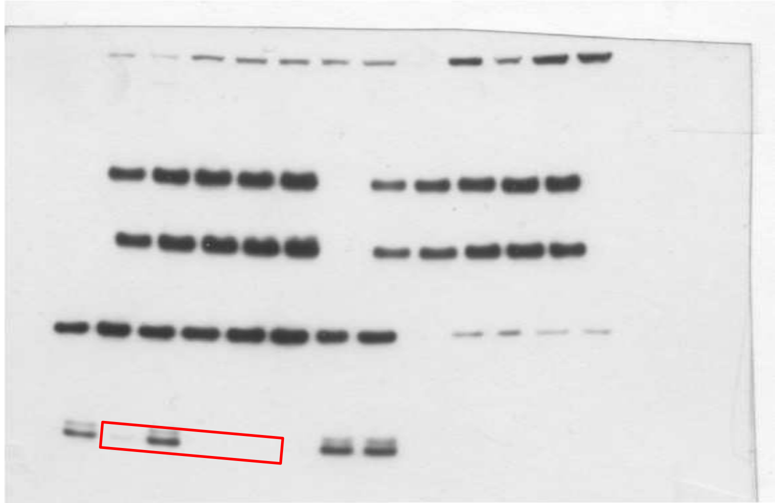

p-ERK1/2

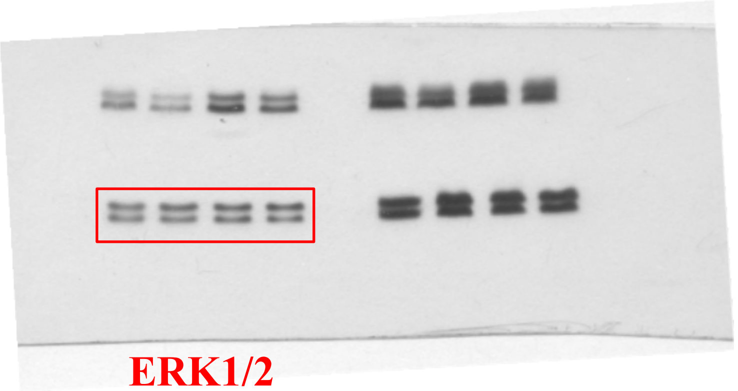

ERK1/2

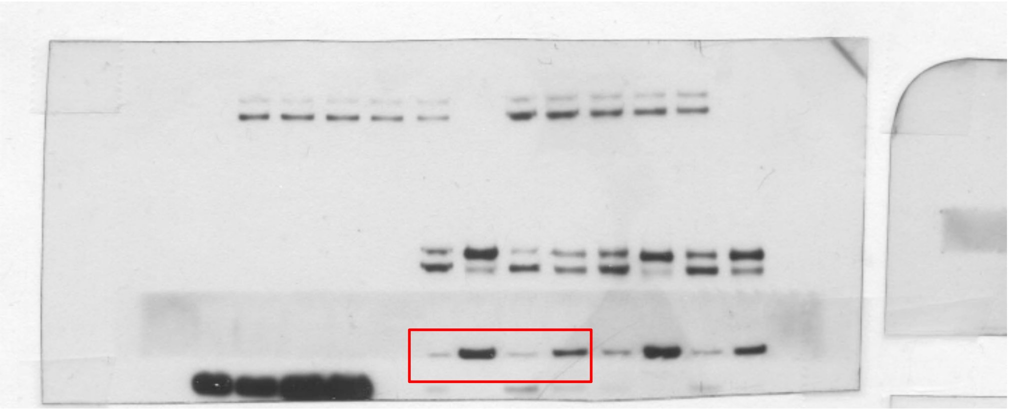

GRP78

Fig. S28

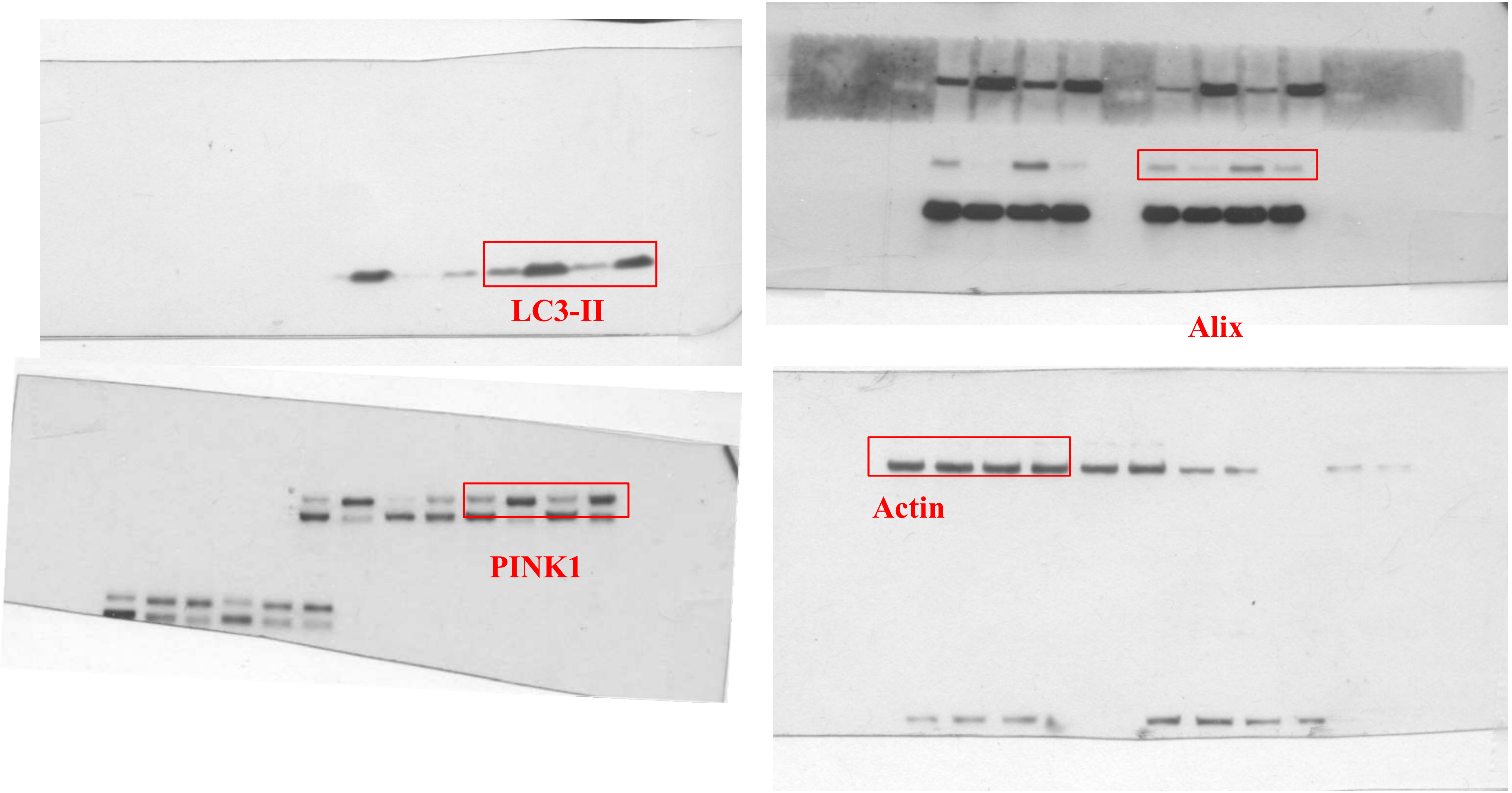

Fig. S29

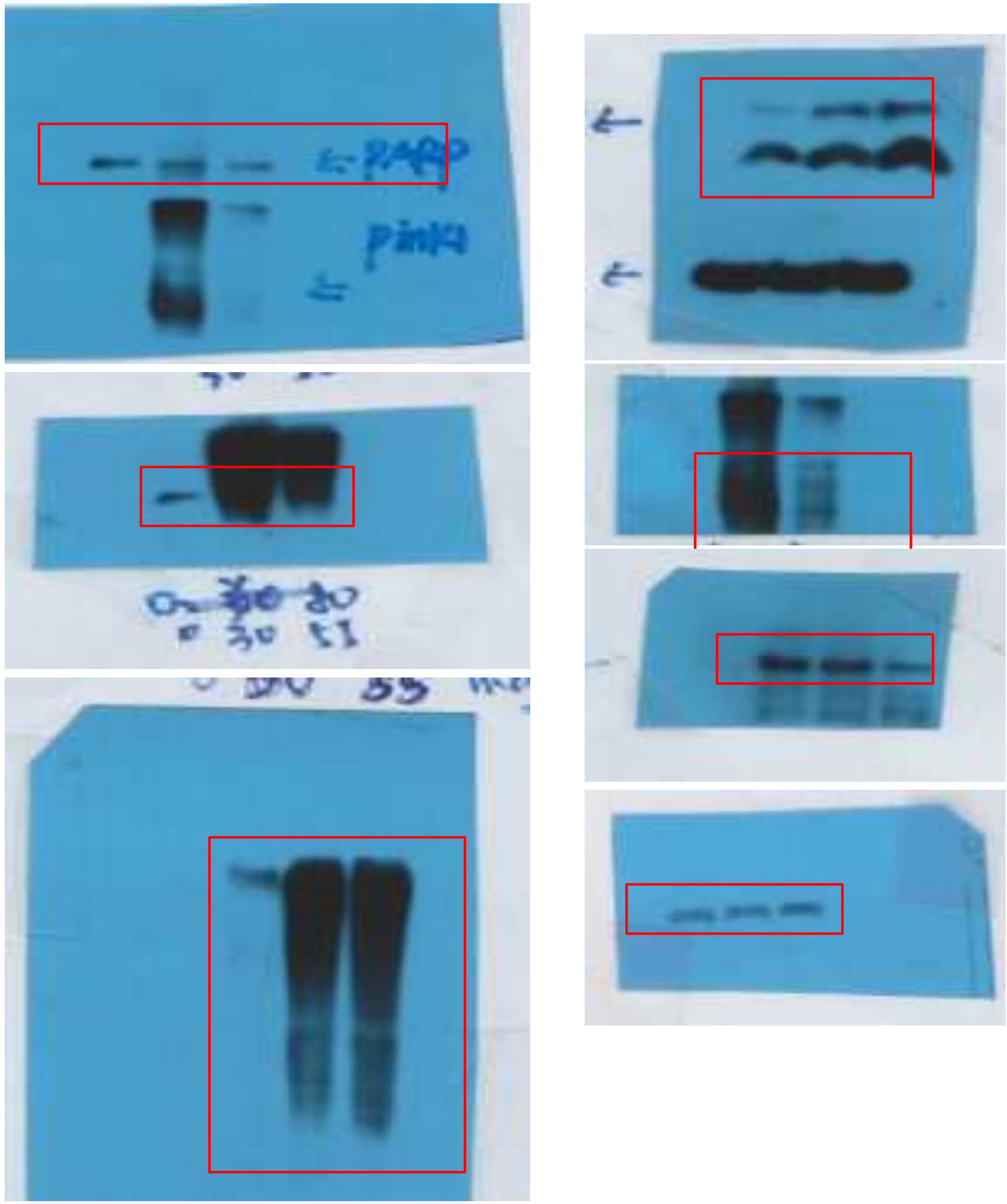

PARP

GRP78

Ubiquitin

LC3-II

PINK-1

Alix

β-Actin

Fig. S30

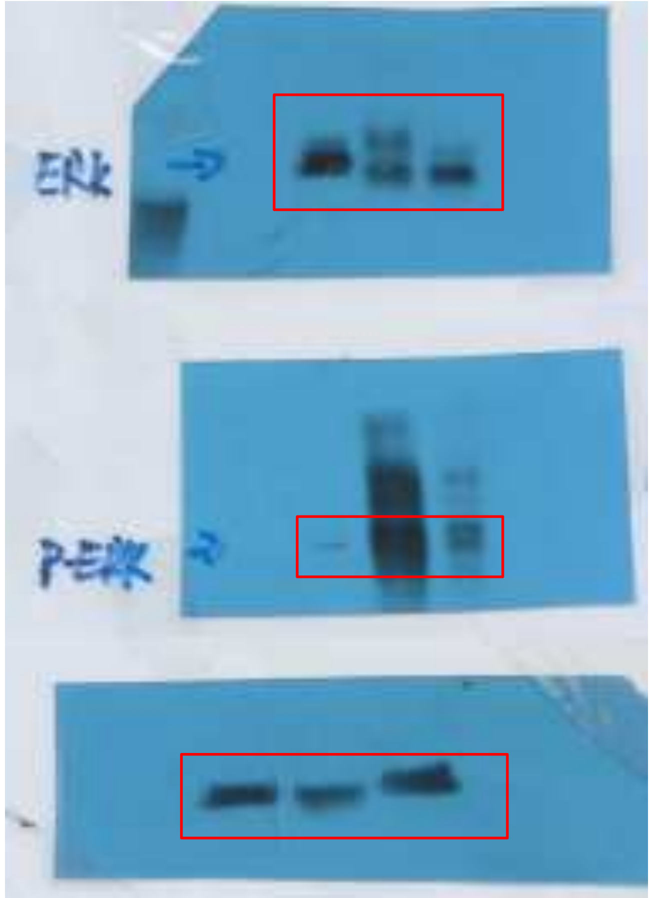

ERK 1/2

p-ERK 1/2

β-Actin

Table S1. The cell viability raw data of MTT assay in MDA-MB-231 cells (mean ± SD, N=3). Cell viability= (OD_{CMR}- OD_{C}) / (OD_{V}- OD_{C})* 100% ; OD_{CMR}: The absorbance data of CMR treatment group; OD_{C}: The absorbance data of blank group; OD_{V}: The absorbance data of control (DMSO) group.

Time (h) \ Con(μM)	0	1	2	3	4	5	6	7	8	9	10
**24**	**100±3**	**100±2**	**100±1**	**100±4**	**98±1**	**93±5**	**77±4**	**52±4**	**40±3**	**31±2**	**25±2**
**48**	**100±4**	**100±3**	**102±3**	**102±7**	**100±4**	**75±4**	**48±2**	**21±3**	**11±1**	**7±1**	**6±0**
**72**	**100±4**	**100±3**	**100±5**	**100±2**	**89±3**	**68±1**	**35±3**	**9±4**	**4±5**	**3±3**	**3±2**

Table S2. The cell viability raw data of MTT assay in PC-3 cells (mean ± SD, N=3). Cell viability= $(OD_{CMR}-OD_{C}) / (OD_{V}- OD_{C})* 100\%$; OD_{CMR}: The absorbance data of CMR treatment group; OD_{C}: The absorbance data of blank group; OD_{V}: The absorbance data of control (DMSO) group.

Con(μM) / Time (h)	0	1	2	3	4	5	6	7	8	9	10
**24**	**100±4**	**97±5**	**92±3**	**88±6**	**83±6**	**76±4**	**71±9**	**62±3**	**48±2**	**35±4**	**28±2**
**48**	**100±8**	**97±9**	**86±9**	**75±7**	**70±11**	**63±8**	**54±8**	**41±2**	**24±3**	**20±3**	**15±1**
**72**	**100±5**	**86±6**	**79±4**	**71±3**	**66±5**	**58±5**	**41±3**	**31±4**	**14±7**	**6±2**	**0±0**

Table S3. The cell viability raw data of MTT assay in LNCaP cells (mean ± SD, N=3). Cell viability= $(OD_{CMR}-OD_{C})$ / $(OD_{V}- OD_{C})$* 100% ; OD_{CMR}: The absorbance data of CMR treatment group; OD_{C}: The absorbance data of blank group; OD_{V}: The absorbance data of control (DMSO) group.

Con(μM) / Time (h)	0	1	2	3	4	5	6	7	8	9	10
**24**	**100±8**	**97±10**	**98±2**	**98±4**	**93±8**	**91±11**	**89±4**	**87±3**	**83±8**	**80±2**	**73±1**
**48**	**100±5**	**98±8**	**95±8**	**93±10**	**78±6**	**71±8**	**66±10**	**58±4**	**43±11**	**42±13**	**40±11**
**72**	**100±7**	**97±1**	**86±2**	**81±4**	**70±2**	**66±5**	**58±10**	**48±2**	**30±5**	**2±2**	**0±0**

Figure S1. (A) Evaluation of CMR inhibition effects in RWPE-2 normal prostate cells**. (B)** The picture of three different non-overlapped regions after CMR treatment for 24 hr, 48 hr and 72 hr in PC-3 and MDA-MB-231.

Figure S2. CMR induced vacuolization in PC-3 and MDA-MB-231 cell lines but not in LNCaP cells. Immunofluorescent images following a 48 h incubation with 6 µM CMR. Red: Calreticulin (1:300). Blue: DAPI. Bar: 25 µm.

Figure S3. Densitometry bands of western blotting in MDA-MB-231, PC-3 and LNCaP cell lines from **Figure 2A** were analyzed by Image J software. Points: mean; bar: SD. Data are expressed as relative protein expression (treated vs. control). *p<0.05, **p<0.01. All assays were performed in triplicate.

Figure S4. CMR induced apoptosis in MCF-10A. Densitometry bands of western blotting in MCF-10A from **Figure 2A** were analyzed by Image J software. Cells were treated with varying concentrations of CMR for 48 h.Points: mean; bar: SD. Data are expressed as relative protein expression (treated vs. control). *p<0.05, **p<0.01. All assays were performed in triplicate.

Figure S5. Densitometry bands of western blotting in MDA-MB-231 and LNCaP cell lines from **Figure 3A-C** were analyzed by Image J software. (**A**) Densitometry bands of western blotting from **Figure 3A.** (**B**) Densitometry bands of western blotting from **Figure 3B.** (**C**) Densitometry bands of western blotting from **Figure 3c.** Points: mean; bar: SD. Data are expressed as relative protein expression (treated vs. control). *p<0.05, **p<0.01. All assays were performed in triplicate.

Figure S6. Immunofluorescent images of MDA-MB-231 and LNCaP cells from **Figure 3A and C**. Red: Calreticulin (1:300). Blue: DAPI. Bar: 25 μm.

Figure S7. Active protein synthesis is required for CMR-induced cytoplasmic vacuolation. Analysis of CMR-induced cytoplasmic vacuolation in MDA-MB-231 and PC-3 cells by (A) immunocytochemistry (from **Figure 4A**) and (B) Western blot (described by densitometry bands from **Figure 4B).** Red: Calreticulin (1:300). Blue: DAPI. Bar: 20 μm. $*p<0.05$, $**p<0.01$. All assays were performed in triplicate.

Figure S8. Immunofluorescent images of MDA-MB-231 and PC-3 cells from **Figure 5A**. Red: Calreticulin (1:300). Blue: DAPI. Bar: 20 μm.

Figure S9. ROS detection by flow cytometry using 2',7'-dichlorodihydrofluorescein diacetate (H2DCFDA).

Figure S10. Densitometry bands of western blotting in MDA-MB-231 and PC-3 cell lines from **Figure 5C** were analyzed by Image J software. Points: mean; bar: SD. Data are expressed as relative protein expression (treated vs. control). $*p<0.05$, $**p<0.01$. All assays were performed in triplicate.

Figure S11. Immunofluorescent images of MDA-MB-231 and PC-3 cells from **Figure 6B**. Red: Calreticulin (1:300). Blue: DAPI. Bar: 20 μm.

Figure S12. MAP kinase plays a role in CMR-induced paraptosis. The effect of U0126 (10 μM) on CMR-induced paraptosis in MDA-MB-231was measured by (A) Western blot (described by densitometry bands from **Figure 7A).** and (B) immunocytochemistry. (from **Figure 7B**). Red: Calreticulin (1:300). Blue: DAPI. Bar: 20 μm. **p*<0.05, ***p*<0.01. All assays were performed in triplicate.

Figure S13. Densitometry bands of western blotting in tumor tissue from **Figure 8C** were analyzed by Image J software. Points: mean; bar: SD. Data are expressed as relative protein expression (treated vs. control). **p*<0.05, ***p*<0.01.

Figure S14. CMR inhibits MDA-MB-231 tumor growth in vivo. (**A**) Western blotting analysis of tumor tissue with one group containing three samples. (**B**) Image of tumors of various groups at 21d after cancer cell injection.

Figure S15. CMR causes the same biochemical signals changes in in vivo as in vitro. (**A)** Immunohistochemical staining of all tumor samples using the LC3B antibody (200×). (B) The immunofluorescent images of all tumor sections after treatment. Confocal images were obtained using the 63× oil immersion lens in an Olympus FV1000 confocal microscope. Blue: DAPI. Red: Calpain1 (1:200). Green: β-Actin (1:200). (**C**) H.E. staining of all tumor samples (200×).

Figure S16. Maximum tolerated dose (MTD) assay was conducted in nude mice. Female and male nude mice were randomized into four groups (n = 4, female and male mice each half) that received 30 mg/kg CMR, 55 mg/kg CMR, 80 mg/kg CMR or vehicle (10% DMSO, 20%

polyethylene glycol and 5% Tween-80 in saline). All treatments were administered by intraperitoneal injection once daily for the entire study and totally for two weeks. 80 mg/kg CMR-treated mice died after a week. 30 mg/kg CMR and 55mg/kg CMR had no significant change in body weight.

Figure S17, Figure S18, Figure S19, Figure S20. The original images of blots in Figure 2A. ECL films (GE, Hyperfilm ECL 5x7 inches) were used to get the blots.

Figure S21. The original images of blots in Figure 3A. ECL films (GE, Hyperfilm ECL 5x7 inches) were used to get the blots.

Figure S22. The original images of blots in Figure 3B. ECL films (GE, Hyperfilm ECL 5x7 inches) were used to get the blots.

Figure S23. The original images of blots in Figure 3C. ECL films (GE, Hyperfilm ECL 5x7 inches) were used to get the blots.

Figure S24. The original images of blots in Figure 4B. ECL films (GE, Hyperfilm ECL 5x7 inches) were used to get the blots.

Figure S25, Figure S26. The original images of blots in Figure 5C. ECL films (GE, Hyperfilm ECL 5x7 inches) were used to get the blots.

Figure S27, Figure S28. The original images of blots in Figure 7A. ECL films (GE, Hyperfilm ECL 5x7 inches) were used to get the blots.

Figure S29, Figure S30. The original images of blots in Figure 8C. X-OMAT BT films (Carestream) were used to get the blots.
